# A fluorescent probe to simultaneously detect both O-GlcNAcase and phosphatase

**DOI:** 10.3389/fchem.2023.1133018

**Published:** 2023-03-01

**Authors:** Jihyeon Boo, Jongwon Lee, Young-Hyun Kim, Chang-Hee Lee, Bonsu Ku, Injae Shin

**Affiliations:** ^1^ Department of Chemistry, Yonsei University, Seoul, Republic of Korea; ^2^ Disease Target Structure Research Center, Korea Research Institute of Bioscience and Biotechnology (KRIBB), Daejeon, Republic of Korea

**Keywords:** fluorescent probe, coumarin, rhodol, enzyme, O-GlcNAcase, phosphatase

## Abstract

O-GlcNAc modification of proteins often has crosstalk with protein phosphorylation. These posttranslational modifications are highly dynamic events that modulate a wide range of cellular processes. Owing to the physiological and pathological significance of protein O-GlcNAcylation and phosphorylation, we designed the fluorescent probe, βGlcNAc-CM-Rhod-P, to differentially detect activities of O-GlcNAcase (OGA) and phosphatase, enzymes that are responsible for these modifications. βGlcNAc-CM-Rhod-P was comprised of a βGlcNAc-conjugated coumarin (βGlcNAc-CM) acting as an OGA substrate, a phosphorylated rhodol (Rhod-P) as a phosphatase substrate and a piperazine bridge. Because the emission wavelength maxima of CM and Rhod liberated from the probe are greatly different (100 nm), spectral interference is avoided. The results of this study revealed that treatment of βGlcNAc-CM-Rhod-P with OGA promotes formation of the GlcNAc-cleaved probe, CM-Rhod-P, and a consequent increase in the intensity of fluorescence associated with free CM. Also, it was found that exposure of the probe to phosphatase produces a dephosphorylated probe, βGlcNAc-CM-Rhod, which displays strong fluorescence arising from free Rhod. On the other hand, when incubated with both OGA and phosphatase, βGlcNAc-CM-Rhod-P was converted to CM-Rhod which lacked both βGlcNAc and phosphoryl groups, in conjunction with increases in the intensities of fluorescence arising from both free CM and Rhod. This probe was employed to detect activities of OGA and phosphatase in cell lysates and to fluorescently image both enzymes in cells. Collectively, the findings indicate that βGlcNAc-CM-Rhod-P can be utilized as a chemical tool to simultaneously determine activities of OGA and phosphatase.

## Introduction

Based on the results of the human genome project (Yang et al., 2016; Aebersold et al., 2018), the number of human proteins encoded by genes is estimated to be around 20,000. This number is increased dramatically by posttranslational modifications with small groups (e.g., Phosphoryl, acetyl or methyl groups) or large biomolecules (e.g., Glycans or Ubiquitin), which give rise to several hundreds of thousands of protein variants (Walsh et al., 2005; Aebersold et al., 2018). Because posttranslational modifications of proteins regulate their activities, structures, interactions, and locations, they play an important role in controlling a broad spectrum of cellular processes (Conibear, 2020). Among these modifications, O-GlcNAc modification of proteins frequently takes place in higher eukaryotes (Hart et al., 2007; Wulff-Fuentes et al., 2021). This glycan modification is a unique type of protein glycosylation in that only a single carbohydrate, N-acetylglucosamine (GlcNAc), is attached to side chains of serine (Ser) or threonine (Thr) residues through the O-linkage. O-GlcNAcylation occurs in cytosolic, nuclear and mitochondrial proteins, and has been suggested to act as a nutrient and stress sensor that modulates various cellular events, including transcription, translation, translocation, cell signaling, and metabolism (Hart et al., 2007; Yang and Qian, 2017). Previous studies have shown that abnormal regulation of protein O-GlcNAc modification is involved in the pathogenesis of diverse human diseases (Slawson and Hart, 2011; Zhu and Hart, 2021). A representative example of diseases caused by dysregulated O-GlcNAcylation is cancer (Ferrer et al., 2016). In addition, aberrant protein O-GlcNAcylation is also associated with type 1 and type 2 diabetes (Ma and Hart, 2013). Furthermore, altered protein O-GlcNAc modification in brain is closely related to the onset of neurodegenerative diseases including Alzheimer’s, Huntington’s and Parkinson’s disorders (Yuzwa, et al., 2012; Lee et al., 2021).

Dynamic cycling of protein O-GlcNAcylation is modulated in a nutrient- and stress-responsive manner by the cooperative action of O-GlcNAcase (OGA) and O-GlcNAc transferase (OGT). While OGA promotes removal of the *ß*-O-GlcNAc moiety from Ser and Thr side chains of cytoplasmic and nuclear proteins, OGT catalyzes the attachment of the GlcNAc monosaccharide to these residues (Dong and Hart, 1994; Gao et al., 2001; Banerjee et al., 2013; Saha et al., 2021). Intriguingly, O-GlcNAc modification of proteins engages in extensive crosstalk with phosphorylation, which is a modification in which a phosphoryl group becomes bonded mainly to the side chains of Ser, Thr and tyrosine (Tyr) of proteins (Hart et al., 2011; van der Laarse et al., 2018). Specifically, O-GlcNAcylation/phosphorylation crosstalk takes place competitively at the same residue within proteins (termed reciprocal crosstalk) or at two different residues that are in close proximity in the protein sequence or are spatially close.

Owing to the pathophysiological importance of protein O-GlcNAcylation and phosphorylation, a critical need exists to develop tools for the detection of enzymes involved in these modifications, in particular, OGA and phosphatase. Among the available methods, fluorescence-based detection is highly attractive and powerful because it is greatly sensitive to analytes, inexpensive and does not require sophisticated instrumentation (Chen et al., 2011; Ko et al., 2011; Vahrmeijer et al., 2013; Yuan et al., 2013; Guo et al., 2014; Chen et al., 2016; Gao et al., 2017; Park et al., 2020; Li et al., 2022). Recently, fluorescent probes have been developed to determine the individual activities of OGA and phosphatase in cells. For example, a coumarin-based activity probe and a coumarin-conjugated fluorescein-based probe have been created to capture and detect OGA in cells (Hyun et al., 2019; Jung et al., 2022). While only two fluorescent probes for OGA have been devised to date, numerous fluorescent probes are available for detection of phosphatases (Li et al., 2012; Li et al., 2012; Li et al., 2017; Liu et al., 2017). Although these fluorescent probes have been successfully applied to monitor OGA and phosphatase individually, analysis of the data arising from concurrent measurements using the different types of probes could be complicated and inaccurate because of their different cell permeability and often spectral interference (Yuan et al., 2012; Chen et al., 2016; Zhang et al., 2016). To overcome this limitation, fluorescent probes that can simultaneously detect multiple analytes have been devised (Kolanowski et al., 2018). In the investigation described below, we designed, prepared, and evaluated the new fluorescent probe, βGlcNAc-CM-Rhod-P, which contains βGlcNAc and phosphoryl groups that have different fluorescence responses to respective OGA and phosphatase. The results of this effort showed that this probe can be employed to determine both OGA and phosphatase activities in cell lysates and live cells.

## Results and discussion

**FIGURE 1:**
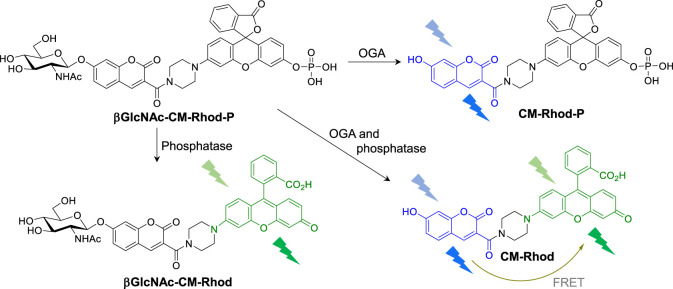
Fluorescence response of βGlcNAc-CM-Rhod-P to O-GlcNAcase (OGA) and phosphatase (see text for detailed explanation).

In considering fluorescent probes for differential detection of OGA and phosphatase, we identified coumarin (CM, *λ*_max,em_ = 440 nm) and rhodol (Rhod, *λ*_max,em_ = 540 nm) as possible fluorescent dyes (Supplementary Figure S1), because their emission maxima are separated by 100 nm and, thus, interference between the responses of the two fluorophores will be minimal. Also, since the emission spectrum of CM overlaps to a certain degree with the absorption spectrum of Rhod (Huang et al., 2015; Li et al., 2018; Bai et al., 2019; Ou et al., 2019), fluorescence resonance energy transfer (FRET) from CM to Rhod would be possible (Park et al., 2019; Park et al., 2020; Park et al., 2021). On this basis, we designed the novel fluorescent probe, βGlcNAc-CM-Rhod-P, which is comprised of a βGlcNAc-conjugated CM serving as the OGA substrate, a phosphate-appended Rhod as the phosphatase substrate and a piperazine bridge (Figure 1).

It was anticipated that βGlcNAc-CM-Rhod-P in which the βGlcNAc and phosphoryl groups are bonded to the hydroxyl groups of respective CM and Rhod would display weak fluorescence. Moreover, we envisaged that treatment of the probe with OGA would lead to a reaction in which the GlcNAc moiety is cleaved from the probe to form CM-Phod-P, thereby increasing the intensity of a fluorescence signal arising from unconjugated CM (Figure 1). On the other hand, addition of phosphatase to the probe would result in cleavage of the phosphoryl group to produce βGlcNAc-CM-Rhod, which would exhibit strong fluorescence from free Rhod. Furthermore, when βGlcNAc-CM-Rhod-P is simultaneously treated with OGA and phosphatase, both βGlcNAc and phosphoryl groups would be cleaved to generate CM-Rhod, an event that would promote increases in the intensities of fluorescence of both CM and Rhod, as well as FRET signals from CM to Rhod.

**SCHEME 1:**
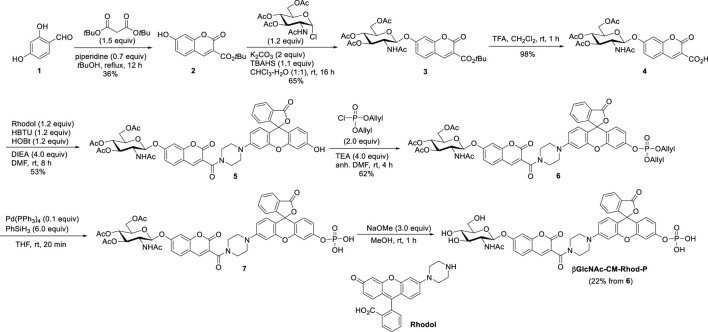
Synthesis of βGlcNAc-CM-Rhod-P.

In line with these design principles, the fluorescent probe βGlcNAc-CM-Rhod-P was prepared by using the sequence depicted in Scheme 1. Briefly, 2,4-dihydroxybenzaldehyde **(1)** was condensed with di-*tert*-butyl malonate to produce adduct **2** that was then subjected to glycosylation with αGlcNAc(OAc)_3_-Cl to form glycoside **3** (Park and Shin, 2007; Hyun et al., 2018). Removal of the *t*-Bu group from **3** generated the corresponding acid **4**, which was reacted with rhodol under amide bond forming conditions to yield **5** (Chen, Pacheco and Takano et al., 2016). The phenolic hydroxyl group in **5** was phosphorylated by reaction with diallyl phosphoryl chloride to form phosphate triester **6** (Li et al., 2014). Finally, the target βGlcNAc-CM-Rhod-P was generated by sequential removal of the allyl and *O*-acetyl groups in **6** using a sequence involving palladium-catalyzed deallylation and treatment with NaOMe, and purification by reversed-phase HPLC (RP-HPLC). In addition, using the pathway shown in Scheme S1, the three analogs βGlcNAc-CM-Rhod, CM-Rhod-P, and CM-Rhod were prepared to confirm the identity of products arising by OGA or/and phosphatase promoted cleavage of βGlcNAc-CM-Rhod-P. All newly synthesized substances were characterized by using NMR and MS methods, and the purities of the final compounds were determined by RP-HPLC.

**FIGURE 2:**
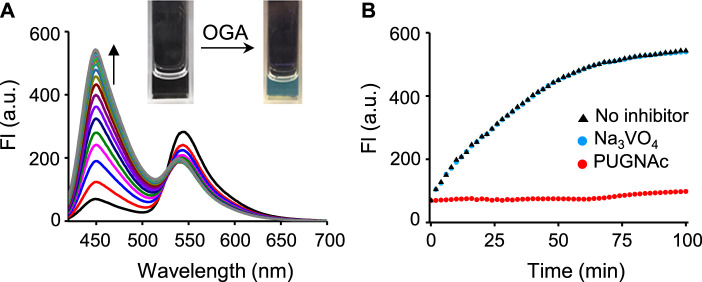
**(A)** Time-dependent change in fluorescence spectra after treatment of βGlcNAc-CM-Rhod-P (10 μM) with OGA (100 nM) in 50 mM Tris buffer (pH 7.4) containing 1% DMSO (λ_ex_ = 400 nm, Δt = 6 min). **(B)** Time-dependent fluorescence response of βGlcNAc-CM-Rhod-P (10 μM) to OGA (100 nM) in the absence and presence of either 50 μM PUGNAc or 1 mM Na_3_VO_4_ (λ_ex_ = 400 nm, λ_em_ = 450 nm, Δt = 2 min).

We next examined the time-dependent fluorescence responses of βGlcNAc-CM-Rhod-P to OGA or/and phosphatase. The results showed that addition of OGA (100 nM) to the probe (10 μM) in Tris buffer (pH 7.4) gives rise to fluorescence from CM at 450 nm (*λ*_ex_ = 400 nm) whose intensity increases until ca. 1 h and then reaches saturation (Figure 2; Supplementary Figure S2). The fluorescence intensity of CM generated under these conditions was increased ca. 10 times. However, when the probe was co-incubated with OGA and its inhibitor PUGNAc (50 μM) (Park and Shin, 2007; Hyun et al., 2018; Hyun et al., 2018), the intensity of the fluorescence arising from CM was not increased. Moreover, in contrast to the OGA inhibitor, the phosphatase inhibitor Na_3_VO_4_ (1 mM) did not affect the fluorescence response of the probe to OGA (Santos et al., 2010; Elkins et al., 2016). The absorption spectra of the probe treated with OGA displayed an increase in absorbance at 400 nm and a slight decrease at 340 nm in a time-dependent manner (Supplementary Figure S3).

**FIGURE 3:**
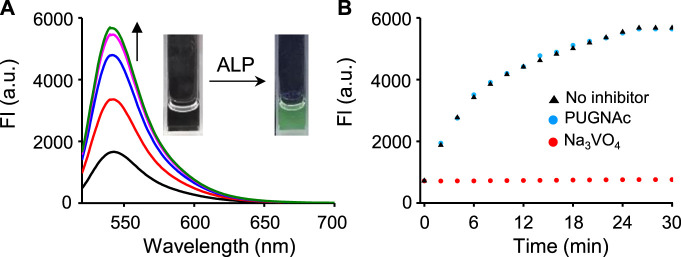
**(A)** Time-dependent change in fluorescence spectra after treatment of βGlcNAc-CM-Rhod-P (10 μM) with ALP (100 nM) in 50 mM Tris buffer (pH 7.4) containing 1% DMSO (λ_ex_ = 510 nm, Δt = 6 min). **(B)** Time-dependent fluorescence response of βGlcNAc-CM-Rhod-P (10 μM) to ALP (100 nM) in the absence and presence of either 50 μM PUGNAc or 1 mM Na_3_VO_4_ (λ_ex_ = 510 nm, λ_em_ = 545 nm, Δt = 2 min).

The fluorescence response of βGlcNAc-CM-Rhod-P to phosphatase was also evaluated. Incubation of the probe (10 μM) with alkaline phosphatase (ALP, 100 nM) in Tris buffer (pH 7.4) resulted in enhancement of the intensity of the fluorescence of Rhod at 545 nm (*λ*_ex_ = 510 nm) up to 25 min (Figure 3). The fluorescence intensity of Rhod produced under these conditions was increased ca. 8 times. However, an increase in fluorescence of Rhod promoted by ALP was not observed when the probe was co-incubated with the inhibitor Na_3_VO_4_ (1 mM). On the contrary, the OGA inhibitor PUGNAc (50 μM) had no influence on the fluorescence response of the probe to ALP. The absorption spectra of the probe treated with ALP showed a time-dependent increase in absorbance at 510 nm (Supplementary Figure S4). We also evaluated the fluorescence responses of βGlcNAc-CM-Rhod-P to other phosphatases. The results showed that protein tyrosine phosphatase receptor type O (PTPRO) and dual-specificity phosphatase 15 (DUSP15) induce fluorescence responses of the probe that are similar to but more rapid than ALP (Supplementary Figure S5, S6).

**FIGURE 4:**
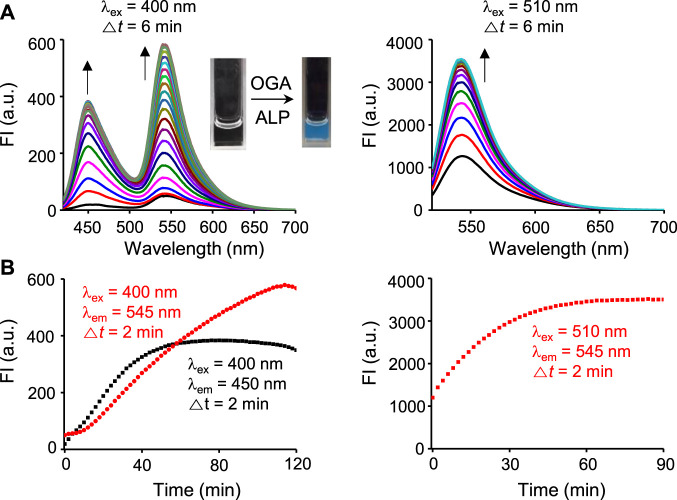
Time-dependent change in fluorescence spectra after treatment of βGlcNAc-CM-Rhod-P (10 μM) with both OGA (100 nM) and ALP (100 nM) in 50 mM Tris buffer (pH 7.4) containing 1% DMSO (upper left: λ_ex_ = 400 nm, Δt = 6 min, upper right: λ_ex_ = 510 nm, Δt = 6 min, lower left: red dot line; λ_ex_ = 400 nm, λ_em_ = 545 nm, Δt = 2 min, black dot line; λ_ex_ = 400 nm, λ_em_ = 450 nm, Δt = 2 min, lower right: λ_ex_ = 510 nm, λ_em_ = 545 nm, Δt = 2 min).

**FIGURE 5:**
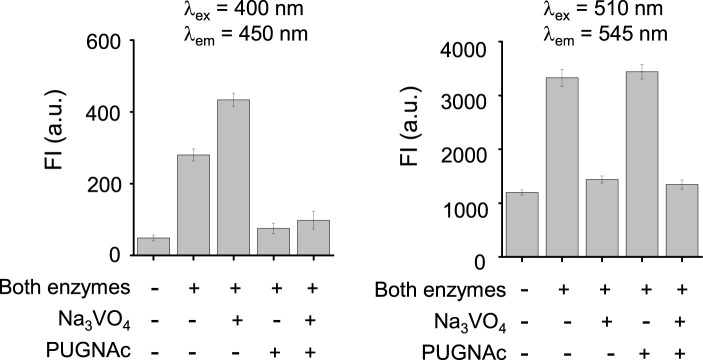
Fluorescence response of βGlcNAc-CM-Rhod-P (10 μM) to OGA (100 nM) and ALP (100 nM) in the absence and presence of 50 μM PUGNAc or/and 1 mM Na_3_VO_4_ (‘+’ means the presence of the indicated substance and ‘−’ does the absence of the indicated substance).

We next assessed the fluorescence response of βGlcNAc-CM-Rhod-P in the presence of both OGA and phosphatase. It was found that addition of both OGA (100 nM) and ALP (100 nM) to the probe (10 μM) results in increases in the intensities of fluorescence associated with CM at 450 nm (*λ*_ex_ = 400 nm) and Rhod at 545 nm (*λ*_ex_ = 510 nm) (Figure 4). Moreover, the signal (545 nm with 400 nm excitation) for FRET from CM to Rhod was enhanced. Inspection of the absorption spectrum of the probe after simultaneous treatment with these enzymes showed that increases in absorbance at 400 nm and 510 nm take place in a time-dependent manner (Supplementary Figure S7). The effect of enzyme inhibitors on the fluorescence response of βGlcNAc-CM-Rhod-P to OGA and phosphatase was also evaluated. When the probe (10 μM) was co-treated with both OGA (100 nM) and ALP (100 nM) in the presence of Na_3_VO_4_ (1 mM), the fluorescence corresponding to CM at 450 nm (*λ*_ex_ = 400 nm) was increased while the fluorescence associated with Rhod at 545 nm (*λ*_ex_ = 510 nm) was not (*λ*_ex_ = 510 nm) (Figure 5). In a corresponding manner, when the probe (10 μM) was co-treated with both enzymes (100 nM) and PUGNAc (50 μM), the fluorescence arising from Rhod was enhanced but that from CM remained unchanged. Furthermore, the results also showed that the intensities of fluorescence arising from both CM and Rhod remain unaltered by treatment of the probe with both OGA and ALP in the presence of both Na_3_VO_4_ and PUGNAc.

We also determined the detection limit of βGlcNAc-CM-Rhod-P for enzymes by conducting titration experiments on the probe with OGA or ALP. When 10 μM βGlcNAc-CM-Rhod-P was treated with various concentrations (0–100 nM) of OGA or ALP, respective fluorescence signals at 450 nm (λ_ex_ = 400 nm) or 545 nm (λ_ex_ = 510 nm) increased in a concentration-dependent fashion (Figure S8). The fluorescence intensity was plotted against the concentration of each enzyme, showing that it is linearly related to the concentration. The regression equations were determined to be ΔF_450 nm_ = 8.18 × [OGA] + 30.9 (*r* ^2^ = 0.99) and ΔF_545 nm_ = 62.5 × [ALP] + 350 (*r* ^2^ = 0.99). The detection limits of the probe for OGA and ALP were calculated to be 4.3 and 3.1 nM, respectively, based on a 3σ/slope method (σ: standard deviation) (Wang et al., 2015). The findings provide evidence that βGlcNAc-CM-Rhod-P can be used to sensitively detect the enzymes.

**FIGURE 6:**
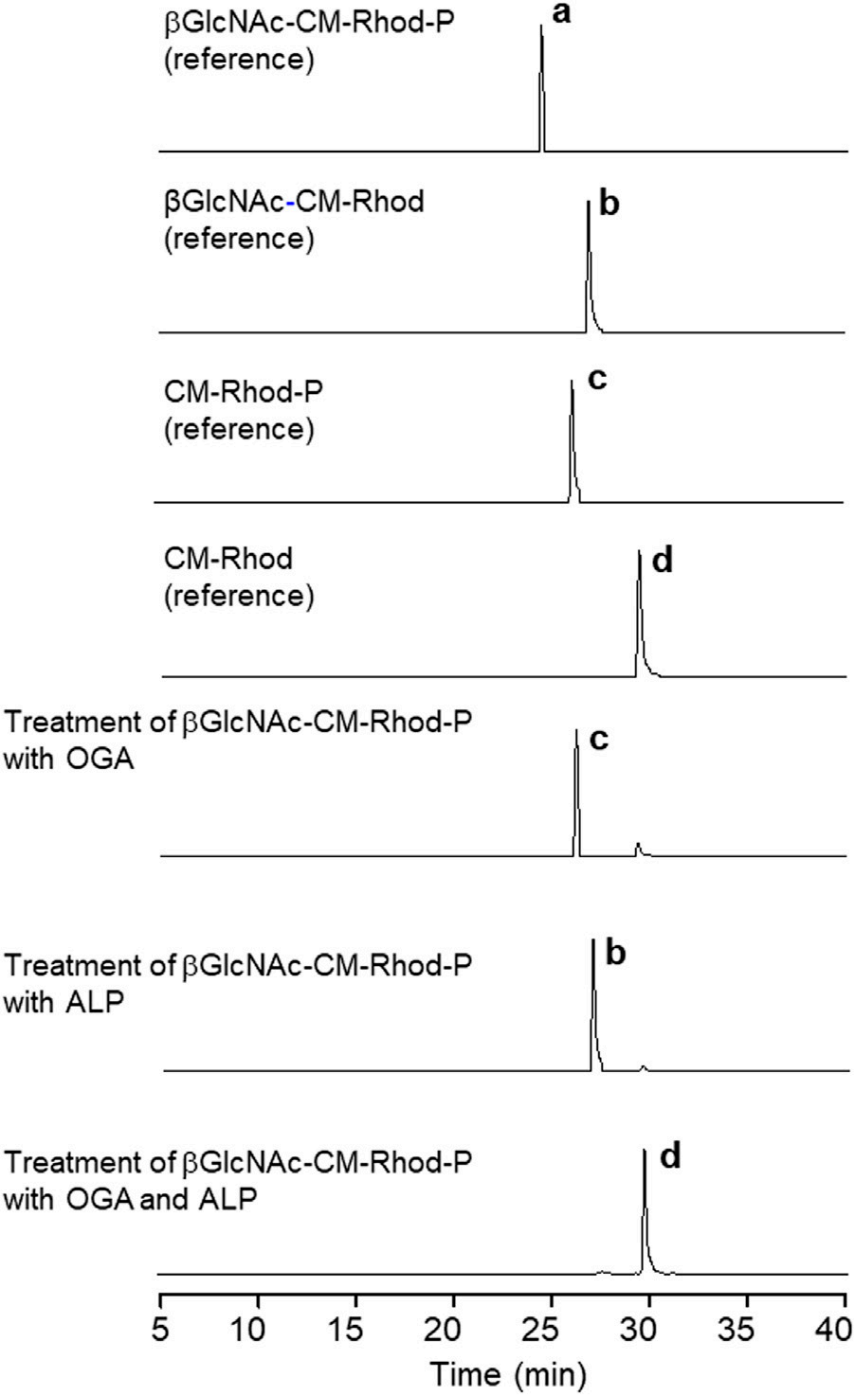
Reversed-phase HPLC analysis of products obtained by treatment of 10 μM βGlcNAc-CM-Rhod-P with OGA (100 nM) or/and ALP (100 nM) (**b** = βGlcNAc-CM-Rhod ([M + H]^+^: m/z = 792.2), **c** = CM-Rhod-P ([M + H]^+^: m/z = 669.1), **d** = CM-Rhod ([M + H]^+^: m/z = 589.1)).

To gain more information about the response of βGlcNAc-CM-Rhod-P to OGA or/and ALP, solutions of the probe (10 μM) were treated with each or both enzymes (100 nM) in the absence and presence of the inhibitors, and then analyzed by RP-HPLC. The HPLC profiles demonstrate that upon treatment of the probe with only OGA, βGlcNAc-CM-Rhod-P completely disappears concomitant with production of CM-Rhod-P (Figure 6). However, co-treatment of the probe with OGA and PUGNAc did not result in formation of CM-Rhod-P (Supplementary Figure S8). It was also found that exposure of the probe to ALP promotes generation of βGlcNAc-CM-Rhod, which does not occur when Na_3_VO_4_ is present. Moreover, the probe was completely converted to CM-Rhod upon treatment with both enzymes. The results of studies with inhibitors revealed that the βGlcNAc-removed (CM-Rhod-P) or phosphoryl group-cleaved product (βGlcNAc-CM-Rhod) is produced when βGlcNAc-CM-Rhod-P is treated with both enzymes and either Na_3_VO_4_ or PUGNAc, respectively. However, βGlcNAc-CM-Rhod-P remained unchanged when it was co-incubated with both enzymes and both inhibitors. The combined results indicate that selective cleavage of the βGlcNAc and phosphoryl groups occurs when βGlcNAc-CM-Rhod-P is treated with OGA and ALP, respectively. Also, the findings support the initial proposal that βGlcNAc-CM-Rhod-P is suitable for simultaneously monitoring activities of both OGA and phosphatase.

**FIGURE 7:**
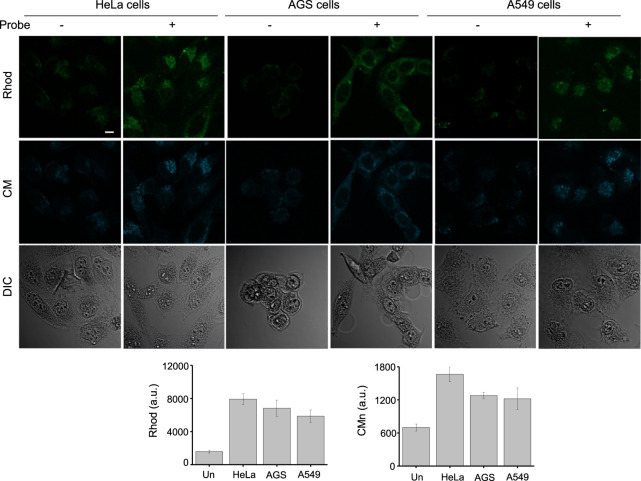
The indicated cells were incubated with 100 μM βGlcNAc-CM-Rhod-P for 18 h. Cell images were obtained by using confocal fluorescence microscopy (scale bar = 10 μm). Graphs show the fluorescence intensity of Rhod (λ_ex_ = 488 nm) and CM (λ_ex_ = 405 nm) in cells (mean ± s.d., *n* = 3).

In the next phase of this investigation, we assessed the use of βGlcNAc-CM-Rhod-P to monitor OGA and phosphatases in live cells. Prior to beginning this study, AGS cells were treated with several non-cytotoxic concentrations (0–100 μM) of βGlcNAc-CM-Rhod-P for 18 h (Supplementary Figure S9). In addition, these cells were incubated with 100 μM βGlcNAc-CM-Rhod-P for several time periods (0–18 h). Analysis of confocal fluorescence microscopy images revealed that AGS cells treated with 100 μM βGlcNAc-CM-Rhod-P for 18 h display substantial fluorescence associated with CM and Rhod when excited at 405 nm and 488 nm, respectively (Supplementary Figures S10, S11). We next explored the application of βGlcNAc-CM-Rhod-P to imaging OGA and phosphatases in other types of cells. For this purpose, HeLa (human cervical cancer cells) and A549 (human lung adenocarcinoma cells) cells along with AGS cells were independently incubated with 100 μM βGlcNAc-CM-Rhod-P for 18 h. Analysis of the intensities of fluorescence arising from CM and Rhod in the treated cells using confocal fluorescence microscopy indicated that OGA and phosphatases activities are dependent on the cell type (Figure 7). Specifically, the activities of both enzymes in HeLa cells are higher than those in AGS and A549 cells, these enzymes in the latter two cells are similar.

Finally, we tested the utility of βGlcNAc-CM-Rhod-P to monitor activities of OGA and phosphatases in cell lysates. In this study, lysates of HeLa, AGS and A549 cells were individually exposed to the probe. Fluorescence intensities corresponding to CM and Rhod dyes in cell lysates were then determined using a fluorescence microplate reader. The results revealed that HeLa cell lysates treated with the probe display higher fluorescence intensities associated with CM and Rhod than do the other cell lysates after treatment (Supplementary Figure S12). These results are consistent with those obtained from experiments using intact cells. Taken together, the findings provide evidence that βGlcNAc-CM-Rhod-P is applicable to both fluorescence imaging of OGA and phosphatases in cells and fluorescence detection of these enzymes in cell lysates.

## Conclusion

Protein O-GlcNAcylation frequently has crosstalk with phosphorylation at the same or two different residues within a protein. Because protein O-GlcNAcylation and phosphorylation are implicated in a wide range of physiological processes and their aberrant modifications cause various human diseases, fluorescent probes for simultaneous detection of both OGA and phosphatase that are crucial for these modifications are in great demand. To date, fluorescent probes that individually measure OGA and phosphatase activities have been developed. However, in many cases, analysis of data obtained using the individual probes for OGA and phosphatase can be both complicating and inaccurate because of different levels of cell penetration and spectral interference. To circumvent this issue, we designed the fluorescent probe βGlcNAc-CM-Rhod-P for monitoring both OGA and phosphatase at the same time. As described above, incubation of βGlcNAc-CM-Rhod-P with phosphatase produced βGlcNAc-CM-Rhod in association with an increase in the intensity of fluorescence arising from Rhod. On the other hand, the fluorescence intensity corresponding to CM was enhanced by production of CM-Rhod-P when the probe was treated with OGA. Moreover, addition of both OGA and phosphatase to the probe led to production of CM-Rhod, thereby increasing the intensities of fluorescence arising from CM and Rhod. The results of cell studies revealed that βGlcNAc-CM-Rhod-P can be employed to fluorescently detect OGA and phosphatase activities in cell lysates and to image these enzymes in cells. It is anticipated that the strategy utilized to design this probe will provide a foundation for creating new probes that possess the ability to concurrently detect two different enzymes.

## Materials and methods

### General

All solvents and chemicals used in the study were purchased from Sigma-Aldrich, Tokyo Chemical Industry (TCI) and Acros in analytical grade, unless particularly mentioned. Alkaline phosphatase (ALP) was purchased from Sigma-Aldrich and other phosphatases (protein tyrosine phosphatase receptor type O (PTPRO) and dual-specificity phosphatase 15 (DUSP15)) were provided by Dr. Bonsu Ku. NMR spectra were recorded on Bruker Avance lll HD 400 and Avance II 400 instruments. High-resolution mass spectrometry data were obtained using an Ultimate 3000 RS-Q-Exactive Orbitrap Plus. UV/VIS absorption spectra were collected on a JASCO V-650 spectrophotometer and fluorescence emission spectra on a JASCO FP-8500 fluorescence spectrophotometer.

### Synthesis of βGlcNAc-CM-Rhod-P

#### Synthesis of compound 2

To a solution of 2,4-dihydroxybenzaldehyde (**1**, 1 g, 7.2 mmol) and di-*tert*-butyl malonate (2.4 mL, 2.3 g, 10.8 mmol) in *tert*-butanol (17 mL) was added piperidine (0.5 mL, 431 mg, 5.06 mmol) with stirring at room temperature. The mixture was stirred at reflux for 12 h and then cooled to room temperature. The mixture was concentrated under reduced pressure, and the residue was subjected to flash column chromatography using hexane/ethyl acetate (v/v, 4:1) as the eluent to give **2** (700 mg) as a pale yellow solid: yield 36%. ^1^H NMR (400 MHz, DMSO-*d*_6_) *d* 8.55 (s, 1 H), 7.72 (d, 1 H, *J* = 11.2 Hz), 6.83 (dd, 1 H, *J* = 11.4, 3.2 Hz), 6.71 (d, 1 H, *J* = 2.8 Hz), 1.51 (s, 9 H). ^13^C NMR (100 MHz, DMSO-*d*_6_) *d* 164.1, 162.2, 157.0, 156.6, 148.5, 131.8, 114.0, 113.5, 110.3, 101.8, 81.2, 27.8. High-resolution mass spectrometry (ESI-MS, m/z): [M + Na]^+^ calcd. for [C_14_H_14_O_5_ + Na]^+^ 285.0739; found 285.0731.

#### Synthesis of compound 3

To a solution of **2** (600 mg, 2.28 mmol) in 1:1 of chloroform and water (12 mL) was sequentially added 2-acetamido-3,4,6-tri-O-acetyl-2-deoxy-*α*-D-glucopyranosyl chloride (αGlcNAc(OAc)_3_-Cl, 1 g, 2.74 mmol), tetrabutylammonium hydrogen sulfate (TBAHS, 855 mg, 2.5 mmol) and K_2_CO_3_ (632 mg, 4.57 mmol) with stirring at room temperature. After stirring for 16 h, the mixture was diluted with dichloromethane and washed sequentially with saturated NH_4_Cl solution, water and brine. The organic layer was dried over anhydrous Na_2_SO_4_, filtered and concentrated under reduced pressure. The residue was subjected to flash column chromatography using hexane/ethyl acetate (v/v, 3:1) as the eluent to give **3** (890 mg) as a white solid: yield 65%. ^1^H NMR (400 MHz, CDCl_3_) *d* 8.30 (s, 1 H), 7.45 (d, 1 H, *J* = 8.8 Hz), 7.00 (s, 1 H), 6.94 (d, 1 H, *J* = 8.4 Hz), 6.58 (d, 1 H, *J* = 9.2 Hz), 5.63 (d, 1 H, *J* = 8.0 Hz), 5.49 (t, 1 H, *J* = 9.2 Hz), 5.13 (t, 1 H, *J* = 9.6 Hz), 4.32–4.07 (m, 4 H), 2.06 (t, 9 H *J* = 12.4 Hz), 1.94 (s, 3 H), 1.57 (s, 9 H). ^13^C NMR (100 MHz, CDCl_3_) *d* 171.1, 170.7, 170.7, 169.5, 161.9, 157.3, 156.7, 147.9, 130.6, 116.0, 115.4, 112.8, 102.8, 97.5, 82.8, 72.2, 72.1, 68.5, 62.0, 54.0, 28.1, 23.2, 20.7, 20.7, 20.6. High-resolution mass spectrometry (ESI-MS, m/z): [M]^−^ calcd. for [C_28_H_33_NO_13_]^−^ 590.1874; found 590.1874.

#### Synthesis of compound 4

A mixture of **3** (600 mg, 1.01 mmol) and trifluoroacetic acid (TFA, 2.5 mL) in dichloromethane (7.5 mL) was stirred for 1 h at room temperature. The mixture was concentrated under reduced pressure to give **4** (531 mg) as a white solid: yield 98%. The crude product was used for the next reaction without further purification. ^1^H NMR (400 MHz, DMSO-*d*_6_) *d* 8.18 (d, 1 H, *J* = 8.8 Hz), 8.08 (s, 1 H), 7.67 (d, 1 H, *J* = 8.8 Hz), 7.04 (s, 1 H), 6.93 (d, 1 H, *J* = 8.4 Hz), 5.48 (d, 1 H, *J* = 8.4 Hz), 5.21 (t, 1 H, *J* = 9.6 Hz), 4.93 (t, 1 H, *J* = 9.6 Hz), 4.20–4.25 (m, 2 H), 4.04–4.18 (m, 2 H), 2.01 (d, 6 H, *J* = 3.2 Hz), 1.95 (s, 1 H), 1.78 (s, 1 H). ^13^C NMR (100 MHz, DMSO-*d*_6_) *d* 170.1, 169.8, 169.8, 169.5, 160.3, 158.5, 158.2, 155.3, 130.9, 118.8, 115.8, 113.9, 102.8, 97.2, 72.5, 71.1, 68.4, 61.7, 53.1, 22.7, 20.5, 20.4. Mass spectrometry (ESI-MS, m/z): [M + H]^+^ calcd. For [C_24_H_25_NO_13_ +H]^+^ 536.1; found 536.6. High-resolution mass spectrometry (ESI-MS, m/z): [M + H]^+^ calcd. For [C_24_H_24_NO_13_ + H]^+^ 534.1253; Found 534.1255.

#### Synthesis of compound 5

A mixture of **4** (320 mg, 0.59 mmol), rhodol (287 mg, 0.71 mmol, see Supporting Information for its synthesis), 2-(1H-benzotriazol-1-yl)-1,1,3,3-tetramethyluronium hexafluorophosphate (HBTU, 272 mg, 0.71 mmol), 1-hydroxybenzotriazole (HOBt, 97 mg, 0.71 mmol) and diisopropylethylamine (DIEA, 0.39 mL, 309 mg, 2.39 mmol) in *N,N′*-dimethylformamide (3 mL) was stirred for 8 h at room temperature. The mixture was diluted with ethyl acetate and washed with water and brine. The organic layer was dried over anhydrous Na_2_SO_4_, filtered and concentrated under reduced pressure. The residue was subjected to flash column chromatography using ethyl acetate/methanol (v/v, 70:1) as the eluent to give **5** (293 mg) as a red solid: yield 53%. ^1^H NMR (400 MHz, DMSO-*d*_6_) *d* 8.20 (s, 1 H), 8.16 (s, 1 H), 7.99 (d, 1 H, *J* = 7.6 Hz), 7.80–7.69 (m, 3 H), 7.25 (d, 1 H, *J* = 7.6 Hz), 7.19 (s, 1 H), 7.04 (d, 1 H, *J* = 8.8 Hz), 6.84 (s, 1 H), 6.76 (d, 1 H, *J* = 8.8 Hz), 6.67 (s, 1 H), 6.57–6.55 (m, 3 H), 5.55 (d, 1 H, *J* = 8.0 Hz), 5.24 (t, 1 H, *J* = 10.4 Hz), 4.96 (t, 1 H, *J* = 9.6 Hz), 4.28–4.19 (m, 2 H), 4.10–4.03 (m, 2 H), 3.73 (s, 2 H), 3.52 (s, 2 H), 3.33 (s, 2 H), 3.25 (s, 2 H), 2.02 (d, 6 H, *J* = 4.4 Hz), 1.96 (s, 3 H), 1.79 (s, 3 H). ^13^C NMR (100 MHz, DMSO-*d*_6_) *d* 170.1, 169.8, 169.7,169.4, 168.8, 163.2, 159.9, 157.9, 155.2, 152.3, 152.2, 151.9, 142.7, 135.5, 130.3, 130.1, 129.2, 128.6, 126.5, 124.8, 124.2, 121.9, 114.2, 113.4, 112.9, 112.1, 109.7, 108.8, 103.1, 102.3, 101.6, 97.1, 72.3, 71.1, 68.3, 61.6, 53.0, 47.8, 47.2, 45.9, 41.1, 22.7, 20.5, 20.5, 20.4. High-resolution mass spectrometry (ESI-MS, m/z): [M + H]^+^ calcd. For [C_48_H_43_N_3_O_16_ + H]^+^ 917.2721; Found 917.2720.

#### Synthesis of compound 6

To a solution of **5** (130 mg, 0.14 mmol) in anhydrous *N,N′*-dimethylformamide (2.3 mL) was added trimethylamine (TEA, 79 μL, 57.3 mg, 0.56 mmol) and diallyl phosphoryl chloride (47 μL, 55 mg, 0.28 mmol, see Supporting Information for its synthesis) with stirring at 0 C under a nitrogen atmosphere. The mixture was warmed to room temperature. After stirring for 4 h at room temperature, the mixture was diluted with ethyl acetate and washed with water and brine. The organic layer was dried over anhydrous Na_2_SO_4_, filtered and concentrated under reduced pressure. The residue was subjected to flash column chromatography using ethyl acetate/methanol (v/v, 90:1) as the eluent to give **6** (95 mg) as a red solid: yield 62%. ^1^H NMR (400 MHz, DMSO-*d*_6_) *d* 8.19 (s, 1 H), 8.16 (d, 1 H*, J* = 9.2 Hz), 8.02 (d, 1 H, *J* = 7.6 Hz), 7.80–7.71 (m, 3 H), 7.30 (d, 1 H, *J* = 7.6 Hz), 7.23 (d, 1 H, *J* = 1.2 Hz), 7.18 (d, 1 H, *J* = 2.0 Hz), 7.03 (dd, 1 H, *J* = 8.6, 2.4 Hz), 6.97 (d, 1 H, *J* = 1.6 Hz), 6.87–6.83 (m, 3 H), 6.60 (d, 1 H, *J* = 8.8 Hz), 5.96–5.94 (m, 2 H), 5.53 (d, 1 H, *J* = 8.4 Hz), 5.39 (s, 1 H), 5.35 (s, 1 H), 5.27–5.20 (m, 3 H), 4.94 (t, 1 H, *J* = 9.6 Hz), 4.67 (t, 3 H, *J* = 4.0 Hz), 4.27–4.18 (m, 2 H), 4.09–4.02 (m, 2 H), 3.73 (s, 2 H), 3.52 (s, 2 H), 3.26 (s, 2 H), 2.01 (d, 6 H, *J* = 4.8 Hz), 1.95 (s, 3 H), 1.78 (s, 3 H). ^13^C NMR (100 MHz, CD_3_OD) *d* 172.2, 171.8, 171.3, 171.1, 165.9, 162.1, 159.8, 156.9, 154.1, 153.5, 153.4, 153.0, 144.6, 136.8, 133.4, 133.3, 131.5, 131.4, 130.8, 129.7, 127.7, 126.0, 125.2, 122.8, 119.4, 117.9, 117.2, 115.7, 114.9, 113.8, 112.1, 110.1, 109.7, 104.6, 103.3, 99.0, 84.2, 73.7, 73.3, 70.6, 70.5, 70.0, 63.2, 55.3, 47.8, 42.9, 22.8, 20.8, 20.6.^31^P NMR (162 MHz, CD_3_OD) *d* 6.60. High-resolution mass spectrometry (ESI-MS, m/z): [M + H]^+^ calcd. For [C_54_H_52_N_3_O_19_P + H]^+^ 1078.3011; found 1078.3011.

### Synthesis of βGlcNAc-CM-Rhod-P

To a solution of **6** (108 mg, 0.1 mmol) and Pd(PPh_3_)_4_ (17 mg, 0.01 mmol) in anhydrous tetrahydrofuran (THF, 3 mL) was added phenylsilane (74.1 μL, 65.1 mg, 0.6 mmol) with stirring at room temperature under an argon atmosphere. After stirring for 20 min, the mixture was diluted with dichloromethane, filtered and washed with dichloromethane. The solvent was removed under reduced pressure to give **7** (100 mg) as a red solid: yield 99%. The crude product was used for the next reaction without further purification. The mixture of crude **7** (100 mg, 0.1 mmol) and sodium methoxide (0.5 M in methanol, 1.0 mL, 0.5 mmol) in methanol (2 mL) was stirred for 1 h at room temperature. After neutralization with Amberite IR-120 (H^+^) ion exchange resins, the mixture was filtered and the resins were washed with methanol thoroughly. The solvent was removed under reduced pressure. The residue was purified by RP-HPLC to give *ß*-GlcNAc-CM-Rhod-P (10 mg) as a red solid: yield 22%. ^1^H NMR (400 MHz, DMSO-*d*_*6*_) *d* 8.18 (s, 1 H), 7.98 (d, 1 H, *J* = 6.8 Hz), 7.87 (d, 1 H, *J* = 9.2 Hz), 7.78–7.68 (m, 3 H), 7.26 (d, 1 H, *J* = 7.2 Hz), 7.20 (s, 1 H), 7.06 (s, 1 H), 6.98 (dd, 1 H, *J* = 8.4, 2.0 Hz), 6.90 (d, 1 H, *J* = 8.8 Hz), 6.83–6.76 (m, 2 H), 6.67 (d, 1 H, *J* = 8.0 Hz), 6.57 (d, 1 H, *J* = 8.8 Hz), 5.16 (d, 1 H, *J* = 8.4 Hz), 3.74–3.61 (m, 4 H), 3.50–3.42 (m, 7 H), 3.30 (s, 2 H), 3.23–3.16 (m, 4 H), 1.81 (s, 3 H). ^13^C NMR (100 MHz, DMSO-*d*_6_) *d* 169.5, 168.8, 163.2, 160.9, 158.0, 155.3, 154.1, 152.5, 152.3, 151.7, 151.4, 142.9, 135.8, 130.3, 128.9, 128.6, 126.0, 124.8, 124.1, 121.5, 116.6, 116.6, 114.3, 113.8, 113.1, 112.3, 108.4, 107.9, 103.1, 101.6, 98.7, 82.6, 77.4, 74.0, 70.2, 60.7, 55.3, 47.7, 47.2, 45.9, 41.1, 23.2.^31^P NMR (162 MHz, DMSO-*d*_6_) *d* −5.80. High-resolution mass spectrometry (ESI-MS, m/z): [M + Na]^+^ calcd. For [C_42_H_38_N_3_O_16_P + Na]^+^ 894.1888; found 894.1880.

## Data Availability

The original contributions presented in the study are included in the article/Supplementary Material, further inquiries can be directed to the corresponding author.
