# Supplementary material for: A fluorescent probe to simultaneously detect both O-GlcNAcase and phosphatase
Source: Front Chem. 2023 Mar 1;11:1133018. doi: 10.3389/fchem.2023.1133018 (PMC10015443; doi:10.3389/fchem.2023.1133018)
Supplement: Supplementary file 1 [file DataSheet1.docx]

Supplementary Material

A fluorescent probe to simultaneously detect both O-GlcNAcase and phosphatase

Jihyeon Boo^1^, Jongwon Lee^1^, Young-Hyun Kim^1^, Chang-Hee Lee^1^, Bonsu Ku^2^, and Injae Shin^1,*^

^1^Department of Chemistry, Yonsei University, Seoul 03722, Republic of Korea.

^2^Disease Target Structure Research Center, Korea Research Institute of Bioscience and Biotechnology (KRIBB), Daejeon 34141, Republic of Korea.

*** Correspondence:** injae@yonsei.ac.kr

**Table of Contents**

1. **Synthesis and Supplementary Schemes** **3**
2. **HPLC profiles of synthesized probes 7**
3. **Methods for *in vitro* Study 7**
4. **Cell study 9**
5. **Supplementary Figures11**
6. **NMR spectra 24**
7. **References 43**

**1. Synthesis and Supplementary Schemes**

**Scheme S1.** Synthesis of CM-Rhod, βGalNAc-CM-Rhod and CM-Rhod-P.

**CM-Rhod.** To a stirred solution of Rhod (233.1 mg, 0.58 mmol, see below for the synthesis of this compound), 7-hydroxycoumarin-3-carboxylic acid (100 mg, 0.48 mmol) , 2-(1H-benzotriazol-1-yl)-1,1,3,3-tetramethyluronium hexafluorophosphate (HBTU, 220.7 mg, 0.58 mmol) and 1-hydroxybenzotriazole (HOBt, 78.6 mg, 0.58 mmol) in anhydrous DMF (4 mL) was added diisopropylethylamine (DIEA, 0.16 mL, 125.3 mg, 0.97 mmol) at room temperature under a nitrogen atmosphere. After stirring for 8 h, the mixture was diluted with EtOAc and washed with water and brine. The organic layer was dried over anhydrous Na_2_SO_4_, filtered and concentrated under reduced pressure. The residue was purified by flash column chromatography (CH_2_Cl_2_ : MeOH = 80:1) to afford **CM-Rhod** as a red solid in 55% yield (158 mg): ^1^H NMR (400 MHz, DMSO-*d*_6_) δ 8.11 (s, 1H), 7.98 (d, 1 H, *J* = 7.6 Hz), 7.80-7.70 (m, 2 H), 7.59 (dd, 1 H, *J* = 8.6, 2.0 Hz), 7.25 (d, 1 H, *J* = 7.6 Hz), 6.84-6.82 (m, 2 H), 6.82-6.74 (m, 2 H), 6.68 (s, 1 H), 6.61 (t, 3 H, *J* = 4.8 Hz), 3.72 (s, 2 H), 3.49 (s, 2 H), 3.32 (s, 2 H), 3.25 (s, 2 H); ^13^C NMR (100 MHz, DMSO-*d*_6_) δ 168.3, 163.0, 162.0, 159.1, 157.6, 155.3, 151.9, 151.7, 151.5, 151.4, 143.0, 135.1, 130.0, 129.6, 128.6, 128.0, 125.8, 124.1, 123.5, 118.9, 113.3, 112.1, 111.5, 110.2, 109.2, 108.2, 101.8, 101.6, 101.0, 47.3, 46.6, 45.5, 40.6. High-resolution mass spectrometry (ESI-MS, m/z): [M + H]^+^ calcd. for [C_34_H_24_N_2_O_8_ + H]^+^ 589.1611; found 589.1610.

**βGlcNAc-CM-Rhod.** To a stirred solution of **5** (70 mg, 0.09 mmol) in MeOH (1 mL) was added NaOMe (0.5 M in methanol, 0.18 mL, 0.08 mmol) at room temperature. The mixture was stirred for 1 h. After neutralization with Amberite IR-120 (H^+^) ion exchange resins, the mixture was filtered and the resins were washed with MeOH thoroughly. The solvent was removed under reduced pressure to give **βGlcNAc-CM-Rhod** as a red solid in 98% yield (60 mg): ^1^H NMR (400 MHz, CD_3_OD) δ 8.08 (s, 1 H), 8,00 (d, 1 H, *J* = 7.2 Hz), 7.74-7.62 (m, 3 H), 7.19 (d, 1 H, *J* = 7.6 Hz), 7.07 (s, 1 H), 7.04 (d, 1 H, *J* = 8.8 Hz), 6.84 (s, 1 H), 6.78-6.67 (m, 2 H), 6.62 (s, 1 H), 6.52 (d, 1 H, *J* = 8.8 Hz), 5.22 (d, 1 H, *J* = 8.4 Hz), 4.00-3.86 (m, 5 H), 3.74 (d, 1 H, *J* = 5.6 Hz), 3.71 (d, 1 H, *J* = 5.6 Hz), 3.64-3.54 (m, 5 H), 3.46-3.42 (m, 3 H), 3.36 (s, 2 H), 1.99 (s, 3 H); ^13^C NMR (100 MHz, DMSO-*d*_6_) δ 169.4, 168.8, 163.2, 160.9, 157.9, 155.2, 152.7, 152.4, 152.1, 142.9, 134.9, 130.3, 130.0, 129.3, 128.7, 127.8, 125.2, 124.6, 121.5, 114.3, 113.0, 112.0, 110.0, 110.0, 109.2, 103.1, 102.4, 101.3, 98.7, 77.9, 73.9, 70.2, 60.6, 55.3, 47.7, 47.1, 45.9, 41.1, 23.1. High-resolution mass spectrometry (ESI-MS, m/z): [M + H]^+^ calcd. for [C_38_H_28_N_2_O_10_ + H]^+^ 792.2404; found 792.2401.

**Compound 9.** To a stirred solution of **8** (180 mg, 0.35 mmol) in anhydrous CH_2_Cl_2_ (4 mL) was added diisopropylethylamine (DIEA, 0.25 mL, 185.9 mg, 1.4 mmol) and diallyl phosphoryl chloride (0.12 mL, 140.9 mg, 0.71 mmol) at 0 ^o^C under a nitrogen atmosphere. After stirring for 10 min at the same temperature, the mixture was warmed to room temperature. After stirring for 8 h, the mixture was diluted with CH_2_Cl_2_ and washed with water and brine. The organic layer was dried over anhydrous Na_2_SO_4_, filtered and concentrated under reduced pressure. The residue was purified by flash column chromatography (hexane : EtOAc = 3:1) to give **9** as a red solid in 57% yield (136 mg): ^1^H NMR (400 MHz, CDCl_3_) δ 8.01 (d, 1 H, *J* = 7.2 Hz), 7.66-7.61 (m, 2 H), 7.16-7.13 (m, 2 H), 6.87-6.86 (m, 1 H), 6.75-6.70 (m, 2 H), 6.64-6.62 (m, 2 H), 6.00-5.88 (m, 2 H), 5.40-5.34 (m, 2 H), 5.30-5.25 (m, 2 H), 4.67-4.62 (m, 4 H), 3.57 (s, 4 H), 3.21 (s, 4 H), 1.47 (s, 9 H); ^13^C NMR (100 MHz, CDCl_3_) δ 169.5, 154.7, 153.0, 152.3, 152.2, 151.8, 135.2, 132.0, 131.9, 129.9, 129.6, 128.9, 126.7, 125.2, 124.0, 119.1, 119.1, 116.3, 115.9, 115.8, 112.5, 109.3, 108.7, 108.6, 102.5, 82.7, 80.2, 69.2, 69.1, 48.3, 28.5; ^31^P NMR (162 MHz, CDCl3) δ -6.82. High-resolution mass spectrometry (ESI-MS, m/z): [M + H]^+^ calcd. for [C_35_H_37_N_2_O_9_P + H]^+^ 661.2315; found 661.2310.

**Compound 10.** To a stirred solution of **9** (100 mg, 0.15 mmol) in CH_2_Cl_2_ (1 mL) was added TFA (0.4 mL) at 0 ^o^C. After stirring for 1 h at room temperature, the mixture was concentrated under reduced pressure to give **10** as a red solid in 98% yield (82 mg). The crude product was used for the next reaction without further purification: ^1^H NMR (400 MHz, CD_3_OD) δ 7.99 (d, 1 H, *J* = 7.2 Hz), 7.73-7.65 (m, 2 H), 7.16 (s, 1 H), 7.10 (d, 1 H, *J* = 7.6 Hz), 6.92 (d, 1 H *J* = 8.8 Hz), 6.81-6.77 (m, 2 H), 6.71 (d, 1 H, *J* =8.8 Hz), 6.61 (d, 1 H, *J* = 8.4 Hz), 6.00-5.90 (m, 2 H), 5.38 (s, 1 H), 5.34 (s, 1 H), 5.24 (d, 2 H, *J* = 10.4 Hz), 4.66 (t, 4 H, *J* = 6.4 Hz), 3.45 (s, 4 H), 3.31 (s, 4 H); ^13^C NMR (100 MHz, CD_3_OD) δ 169.7, 152.7, 152.1, 152.0, 152.0, 135.5, 131.9, 131.8, 130.1, 129.4, 128.5, 126.2, 124.7, 123.7, 118.1, 116.5, 115.9, 112.7, 109.7, 108.4, 108.4, 102.5, 82.7, 69.3, 69.3, 45.1, 43.0; ^31^P NMR (162 MHz, CDCl_3_) δ -6.84. High-resolution mass spectrometry (ESI-MS, m/z): [M + H]^+^ calcd. for [C_35_H_37_N_2_O_9_P + H]^+^ 661.2315; found 661.2310.

**Compound 11.** To a stirred solution of **10** (163 mg, 0.28 mmol), 7-hydroxy-coumarin-3-carboxylic acid (50 mg, 0.24 mmol), 2-(1H-benzotriazol-1-yl)-1,1,3,3-tetramethyluronium hexafluorophosphate (HBTU, 110.3 mg, 0.24 mmol) and 1-hydroxybenzotriazole (HOBt, 39.3 mg, 0.24 mmol) in anhydrous DMF (2.5 mL) was added diisopropylethylamine (DIEA, 0.16 mL, 125.3 mg, 0.97 mmol) at room temperature under a nitrogen atmosphere. After stirring for 8 h, the mixture was diluted with EtOAc and washed with water and brine. The organic layer was dried over anhydrous Na_2_SO_4_, filtered and concentrated under reduced pressure. The residue was purified by flash column chromatography (CH_2_Cl_2_ : MeOH = 60:1) to give **11** as a red solid in 67% yield (122 mg): ^1^H NMR (400 MHz, CDCl_3_) δ 7.99 (d, 1 H, *J* = 7.6 Hz), 7.86 (s, 1 H), 7.68-7.51 (m, 2 H), 7.27 (d, 1 H, *J* = 9.2 Hz), 7.13 (d, 2 H, *J* = 8.0 Hz), 6.86 (d, 1 H, *J* = 8.8 Hz), 6.75-6.60 (m, 6 H), 5.98-5.88 (m, 2 H), 5.40 (s, 1 H), 5.36 (s, 1 H), 5.28 (d, 2 H, *J* = 10.4 Hz), 4.66 (t, 4 H, *J* = 7.2 Hz), 3.90 (s, 2 H), 3.53 (s, 2 H), 3.31 (d, 4 H, *J* = 26.0 Hz); ^13^C NMR (100 MHz, CDCl_3_) δ 169.6, 164.8, 162.6, 158.8, 156.2, 152.8, 152.5, 152.3, 151.6, 144.9, 135.3, 131.7, 131.6, 130.1, 130.0, 129.6, 129.4, 128.8, 126.6, 125.2, 124.0, 119.4, 119.1, 116.4, 115.8, 114.4, 112.6, 111.1, 109.2, 108.7, 103.1, 102.5, 82.9, 69.5, 69.4, 48.4, 47.9, 46.9, 42.1; ^31^P NMR (162 MHz, CDCl_3_) δ -6.76. High-resolution mass spectrometry (ESI-MS, m/z): [M + H]^+^ calcd. for [C_40_H_33_N_2_O_11_P + H]^+^ 749.1900; found 749.1900.

**CM-Rhod-P.** To a stirred solution of **11** (55 mg, 0.07 mmol) and Pd(PPh_3_)_4_ (8.48 mg, 0.007 mmol) in anhydrous THF (1 mL) was added phenylsilane (54.3 μL, 47.7 mg, 0.44 mmol) at 0 ^o^C under an argon atmosphere. After stirring for 20 min at room temperature, the mixture was concentrated under reduced pressure. The residue was purified by reversed-phase HPLC to give **CM-Rhod-P** as a red solid in 34% yield (17 mg): ^1^H NMR (400 MHz, DMSO-*d*_6_) δ 8.11 (s, 1 H), 7.98 (d, 1 H, *J* = 7.6 Hz), 7.78-7.68 (m, 2 H), 7.58 (d, 1 H, *J* = 8.4 Hz), 7.25 (d, 1 H, *J* = 7.2 Hz), 7.21 (s, 1 H), 6.88-6.83 (m, 2 H), 6.79-6.74 (m, 3 H), 6.59(dd, 2 H, *J* = 28.8, 8.8 Hz), 3.70 (s, 2 H), 3.46 (s, 2 H), 3.29 (s, 2 H), 3.21 (s, 2 H); ^13^C NMR (100 MHz, DMSO-*d*_6_) δ 168.7, 163.5, 162.4, 158.1, 155.8, 152.4, 152.3, 151.7, 151.4, 143.5, 135.7, 130.5, 130.2, 129.0, 128.8, 128.5, 126.0, 124.7, 124.1, 119.5, 116.6, 113.8, 113.7, 112.2, 110.8, 108.3, 107.8, 102.1, 101.5, 82.6, 47.7, 47.1, 45.9, 41.1; ^31^P NMR (162 MHz, DMSO-*d*_6_) δ -6.49. High-resolution mass spectrometry (ESI-MS, m/z): [M + H]^+^ calcd. for [C_34_H_25_N_2_O_11_P + H]^+^ 669.1274; found 669.1272.

**Scheme S2.** Synthesis of rhodol.

**Synthesis of rhodol.** TFA (5.6 mL) was added to 2-(2,4-dihydroxybenzoyl)benzoic acid (565 mg, 2.19 mmol) and 3-(1-piperazinyl) phenol (300 mg, 1.68 mmol) at room temperature. The mixture was heated at reflux for 9 h and then cooled to room temperature. The mixture was diluted with methanol and concentrated under reduced pressure. To a stirred solution of the crude compound in 4 mL methanol was added TEA and Boc_2_O (0.4 mL, 1.68 mmol) at 0 ^o^C. After stirring for 3 h room temperature, the volatile material was removed under reduced pressure. The residue was dissolved into dichloromethane. The organic layer was washed with water and brine. The organic layer was dried over anhydrous Na_2_SO_4_, filtered and concentrated under reduced pressure. The residue was purified by flash column chromatography (CH_2_Cl_2_ : MeOH = 90:1) to give **8** as a red solid in 62% yield (95 mg): ^1^H NMR (400 MHz, CDCl_3_) δ 8.01 (d, 1 H, *J* = 8.0 Hz), 7.62-7.55 (m, 2 H), 7.11 (d, 1 H, *J* = 4.0 Hz), 6.70-6.53 (m, 6 H), 3.53 (s, 4 H), 3.20 (s, 4 H), 1.47 (s, 9 H); ^13^C NMR (100 MHz, CDCl_3_) δ 170.1, 160.0, 154.8, 153.1, 153.0, 151.5, 151.4, 134.6, 129.7, 129.4, 129.0, 127.7, 125.4, 124.5, 113.1, 112.2, 111.1, 110.0, 103.1, 102.0, 80.4, 50.8, 48.0, 28.4. High-resolution mass spectrometry (ESI-MS, m/z): [M + H]^+^ calcd. for [C_29_H_28_N_2_O_6_ + H]^+^ 501.2025; found 501.2022.

To a stirred solution of compound **8** (130 mg, 0.26 mmol) was added TFA (0.7 mL) in 3 mL CH_2_Cl_2_ at 0 ^o^C. The mixture was warmed to room temperature. After stirring for 1 h, the volatile material was removed under reduced pressure. The crude product was used for the next reaction without further purification: ^1^H NMR (300 MHz, CD_3_OD) δ 8.23 (dd, 1 H, *J* = 7.5, 1.2 Hz), 7.86-7.75 (m, 2 H), 7.34 (dd, 1 H, *J* = 6.0, 3.0 Hz), 7.17-7.00 (m, 5 H), 6.87-6.83 (m, 1 H), 3.87-3.84 (m, 4 H), 3.43-3.40 (m, 4 H); ^13^C NMR (75 MHz, CD_3_OD) δ 168.0, 165.3, 156.4, 155.8, 155.5, 133.7, 130.6, 130.5, 130.3, 129.0, 128.5, 127.4, 115.8, 114.6, 113.6, 113.5, 102.1, 99.6, 44.5, 42.8. ESI-MS calcd for C_24_H_20_N_2_O_4_ [M + H]^+^ = 401.4, found 401.6. High-resolution mass spectrometry (ESI-MS, m/z): [M - H]^-^ calcd. for [C_24_H_20_N_2_O_4_ - H]^-^ 399.1345; found 399.1349.

**Scheme S3.** Synthesis of diallyl phosphoryl chloride.

**Synthesis of diallyl phosphoryl chloride.** Methyl dichlorophosphate (2.5 mL, 3.72 g, 25 mmol) in anhydrous pyridine (25 mL) was added to allyl alcohol (8.5 mL, 7.26 g, 125 mmol) at 0 ^o^C. The mixture was warmed to room temperature. After stirring for 9 h, the reaction was quenched by addition of saturated aqueous NaHCO_3_. The aqueous layer was washed with diethyl ether to remove excess allyl alcohol and acidified to pH 1 with concentrated HCl. The aqueous layer was extracted with the mixture of CH_2_Cl_2_ and *t*-BuOH (7:3). The combined organic layers were washed with 1 M HCl and brine, dried over anhydrous Na_2_SO_4_ and filtered. The solvent was removed under reduced pressure to give **12** as a colorless oil in 62% yield (2.89 g). The crude product was used without further purification for the next reaction: ^1^H NMR (400 MHz, CDCl_3_) δ 12.1 (s, 1 H), 6.00-5.86 (m, 2 H), 5.36-5.35 (m, 2 H), 5.32-5.31 (m, 2 H), 5.23-5.20 (m, 2 H), 4.52-4.48 (m, 4 H); ^13^C NMR (100 MHz, CDCl_3_) δ 132.4, 132.3, 117.9, 67.9, 67.9; ^31^P (162 MHz, CDCl_3_) δ 0.81.

To a stirred solution of oxalyl chloride (170 μL, 251 mg, 1.97 mmol) in anhydrous CH_2_Cl_2_ (3 mL) was added anhydrous DMF (5.4 μL, 5 mg, 0.07 mmol) at 0 ^o^C under a nitrogen atmosphere. The mixture was warmed to room temperature. After stirring for 10 min, a solution of **12** (117 mg, 0.66 mmol) in anhydrous CH_2_Cl_2_ (3 mL) was added to the above mixture at room temperature. After stirring for 20 min, the volatile material was removed under reduced pressure to afford diallyl phosphoryl chloride as a pale yellow oil in 62% yield (80 mg). The crude product was used without further purification for the next reaction: ^1^H NMR (400 MHz, CDCl_3_) δ 6.01-5.90 (m, 2 H), 5.45-5.27 (m, 4 H), 4.71-4.65 (m, 4 H); ^13^C NMR (100 MHz, CDCl_3_) δ 130.5, 130.4, 119.1, 69.4, 69.3; ^31^P (162 MHz, CDCl_3_) δ 4.12.

**2. HPLC profiles of synthesized probes**

Analytic RP-HPLC (C18 column, 250 x 4.6 mm; pore size, 5 μM) with a gradient of 5-100% CH_3_CN in water (0.1% TFA) over 45 min (a flow rate; 1 mL/min, detection at 330 nm).

**3. Methods for *in vitro* study**

**Cloning of O-GlcNAcase (BtGH84) from *B. thetaiotaomicron.***  *E. coli* DH5a strain was used for cloning and maintenance of plasmids and BL21(DE3) strain for protein expression. O-GlcNAcase ORF (22-737, BtGH84) was amplified from *B. thetaiotaomicron* KCTC 5723 (VPI-5482, ATCC 29148) genomic DNA template by PCR using i-pfu DNA polymerase (Intron) and a pair of primers 5’-CGCGCGGATCCC AGAATGTTAGTCTGCAACCTCC-3’ and 5’-CCCCGCTCGAGTTATTTCTTCTCTA TAGTCAGA-3’. PCR products were cloned into pET28a to obtain pET28a-BtGH84. The cloned sequence was confirmed by DNA sequencing at Macrogen Company (Korea).

**Expression of His_6_-tagged** **BtGH84.** *E. coli* BL21(DE3) competent cells were transformed with pET28a-BtGH84. A single colony was grown in 10 mL of Luria-Bertani (LB) media containing 50 μg/mL Kanamycin at 37 ^o^C for 14 h with shaking. After incubation, 1 L LB media containing Kanamycin was inoculated with 14 h cell cultures and grown with shaking until the OD600 reached to 0.6. Expression of a His6-tagged fusion protein was induced by incubation with 0.5 mM isopropyl-β-thiogalactopyranoside (IPTG, Bio Basic) for 4 h at 37 ^o^C.

**Purification of His_6_-tagged** **BtGH84.** All purification steps were performed on ice or at 4 ^o^C. The cells were harvested by centrifugation at 5,000 rpm for 10 min at 4 ^o^C and the supernatant was discarded. The cell pellets were re-suspended in an ice cold buffer (20 mM HEPES, 400 mM NaCl, 1 tablet of EDTA-free protease inhibitor cocktail (Roche), pH 7.4) and lysed by treatment with 0.2 mg/mL lysozyme (Sigma) for 1 h at 25 ^o^C or by sonication. Cell debris and inclusion body were removed by centrifugation at 15,000 rpm for 30 min at 4 ^o^C. The supernatant was filtered through 0.45 μm pore nitrocellulose membrane and the filtrate was poured onto HisPur­^TM^ Ni-NTA resin (Thermo) in column at 4 ^o^C. After 30 min, the column was washed with binding buffer and wash buffer (20 mM HEPES, 400 mM NaCl, 50 mM imidazole, pH 7.4). The His_6_-tagged protein was eluted with 10 volumes of elution buffer (20 mM HEPES, 400 mM NaCl, 200 mM imidazole, pH 7.4). The eluted protein was concentrated and exchanged into 20 mM HEPES (pH 7.4) containing 300 mM NaCl using an Amicon centrifugal filter (Merck, 10 KDa). The purified protein was analyzed by SDS-PAGE and visualized by Coomassie Blue staining.

**<**SDS-PAGE of purified *N*-terminal His_6_-tagged BtGH84>

**Fluorescence response of βGlcNAc-CM-Rhod-P to O-GlcNAcase.** O-GlcNAcase (100 nM) was added to 10 μM βGlcNAc-CM-Rhod-P in 50 mM Tris buffer (pH 7.4) containing 1% DMSO in a final volume of 120 μL at the start of the incubation at 37 ^o^C. For inhibition study, O-GlcNAcase (100 nM), pretreated with 50 μM PUGNAc (Biosynth) for 1 h at 37 ^o^C, was added to 10 μM βGlcNAc-CM-Rhod-P in 50 mM Tris buffer (pH 7.4) containing 1% DMSO in a final volume of 120 μL at the start of the incubation at 37 ^o^C. Production of CM-Rhod-P by cleavage of O-GlcNAc from the probe by O-GlcNAcase was detected using a fluorometer (JASCO, FT-8500) with excitation at 400 nm and emission at 450 nm. The reaction was run for 100 min with readings taken every 2 min.

**Fluorescence response of βGlcNAc-CM-Rhod-P to phosphatase.** Alkaline phosphatase (100 nM), protein tyrosine phosphatase receptor-type O (PTPRO, 100 nM) or dual specificity phosphatase 15 (DUSP 15, 100 nM) was separately added to 10 μM βGlcNAc-CM-Rhod-P in 50 mM Tris buffer (pH 7.4) containing 1% DMSO in a final volume of 120 μL at the start of the incubation at 37 ^o^C. For inhibition study, each phosphatase (100 nM), pretreated with 1 mM Na_3_VO_4_ for 1 h at 37 ^o^C, was added to 10 μM βGlcNAc-CM-Rhod-P in 50 mM Tris buffer (pH 7.4) containing 1% DMSO in a final volume of 120 μL at the start of the incubation at 37 ^o^C. Production of βGlcNAc-CM-Rhod by cleavage of phosphate from the probe by phosphatase was detected using a fluorometer with excitation at 510 nm and emission at 545 nm.

**Fluorescence response of βGlcNAc-CM-Rhod-P to O-GlcNAcase and alkaline phosphatase.** O-GlcNAcase (100 nM) and alkaline phosphatase (100 nM) were added to 10 μM βGlcNAc-CM-Rhod-P in 50 mM Tris buffer (pH 7.4) containing 1 % DMSO in a final volume of 120 μL at the start of the incubation at 37 ^o^C. Production of CM-Rhod by action of both enzymes was monitored using a fluorometer with excitation at 400 nm/emission at 450 nm, excitation at 400 nm/emission at 545 nm or excitation at 510 nm/emission at 545 nm for indicated times.

**Measurement of detection limit of the probe for enzymes.** Various concentrations of O-GlcNAcase (10-100 nM) or alkaline phosphatase (10-100 nM) were separately added to 10 μM βGlcNAc-CM-Rhod-P in 50 mM Tris buffer (pH 7.4) containing 1% DMSO in a final volume of 120 μL at 37 ^o^C. After incubation for 100 min (in case of O-GlcNAcase) or 30 min (in case of alkaline phosphatase) at 37 ^o^C, changes in fluorescence intensity were detected using a JASCO FP-8500 fluorimeter (CM: λ_ex_ = 400 nm, λ_em_ = 450 nm; Rhod: λ_ex_ = 510 nm, λ_em_ = 545 nm).

**HPLC analysis of βGlcNAc-CM-Rhod-P treated with enzyme**. βGlcNAc-CM-Rhod-P (10 μM) was individually incubated with O-GlcNAcase (100 nM) or/and alkaline phosphatase (100 nM) in the absence or presence of 50 μM PUGNAc or/and 1 mM Na_3_VO_4_ for 1 h at 37 ^o^C. The solutions were analyzed by using analytical RP-HPLC with a gradient of 5-100% CH_3_CN (0.1% TFA) in water (0.1% TFA) over 45 min (a flow rate; 1 mL/min, detection at 330 nm).

**4. Cell study**

**Cell Culture.** AGS (human gastric adenocarcinoma cells), HeLa (human cervical cancer cells) and A549 (human lung adenocarcinoma cells) cells were cultured in RPMI 1640 media (Gibco) or DMEM media (Gibco) that were supplemented with 10% fetal bovine serum (FBS), 50 units/mL penicillin and 50 units/mL streptomycin. Cells were maintained at 37 ^o^C under a humidified atmosphere containing 5% CO2.

**Cell viability assay.** Cell viability was assessed by using an MTT ((3-(4,5-dimethylthiazol-2-yl)-2,5-diphenyltetrazolium bromide) assay. Cells (5 x 10^3^ cells/100 μL) were plated in triplicate in 96-well plates for 24 h and were incubated with various concentrations of βGlcNAc-CM-Rhod-P in culture media for 24 h. 20 μL of MTT (5 mg/mL, Amresco Life Science) was added to culture media in each well and the mixture was then incubated for 3 h. After removing the culture media containing MTT, 100 μL of DMSO was added and incubated for 30 min for color development. The absorbance at 570 nm was measured using an Infinite® 200 PRO multimode microplate reader (Tecan).

**Cell imaging.** Cells in culture media were incubated with various concentrations of βGlcNAc-CM-Rhod-P for 18 h at 37 ^o^C. The cells were washed three times with Dulbecco's phosphate-buffered saline (DPBS) and imaged by using confocal fluorescence microscopy (Zeiss LSM 800). All cell images were representative of at least three independent experiments.

**Quantitation of fluorescence intensity in cells.** Fluorescence intensities in cells were quantified using the mean region of interest (ROI) tool with the ZEN software.

**Cell lysis experiment.** Cells were lysed by using RIPA buffer (50 mM Tris, 150 mM NaCl, 1% NP-40, pH 7.4) containing 1 tablet of a protease inhibitor cocktail (Roche) on ice for 30 min. The cell lysates were centrifuged for 15 min at 14,000 rpm. The total protein concentration was determined by using the Pierce^TM^ BCA Protein Assay kit (Thermo Scientific^TM^). The supernatant was transferred into a new tube for further analysis or keeping at -80 ^o^C. Cell lysates were incubated with 10 μM βGlcNAc-CM-Rhod-P in 50 mM Tris buffer (pH 7.4) at 37 ^o^C. Fluorescence intensities of FITC and coumarin were measured using an Infinite® 200 PRO multimode microplate reader (coumarin, λ_ex_ = 400 nm, λ_em_ = 450 nm; rhodol, λ_ex_ = 510 nm, λ_em_ = 545 nm).

**5. Supplementary Figures**

**Figure S1.** Normalized absorption and emission spectra of coumarin (CM) and rhodol (Rhod) in 50 mM Tris buffer (pH 7.4) containing 1% DMSO.

**Figure S2.** (a) Absorption and (b) fluorescence emission spectra of CM-Rhod and βGlcNAc-CM-Rhod-P in 50 mM Tris buffer (pH 7.4) containing 1% DMSO.

**Figure S3.** Time-dependent change in absorption spectra after treatment of βGlcNAc-CM-Rhod-P (10 μM) with OGA (100 nM) in 50 mM Tris buffer (pH 7.4) containing 1% DMSO.

**Figure S4.** Time-dependent change in absorption spectra after treatment of βGlcNAc-CM-Rhod-P (10 μM) with ALP (100 nM) in 50 mM Tris buffer (pH 7.4) containing 1% DMSO.

**Figure S5.** (a) Time-dependent change in fluorescence spectra after treatment of βGlcNAc-CM-Rhod-P (10 μM) with protein tyrosine phosphatase receptor type O (PTPRO, 100 nM) in 50 mM Tris buffer (pH 7.4) containing 1% DMSO (λ_ex_ = 510 nm, Δ*t* = 10 sec). (b) Time-dependent fluorescence response of βGlcNAc-CM-Rhod-P (10 μM) to PTPRO (100 nM) in the absence or presence of 1 mM Na_3_VO_4_ (λ_ex_ = 510 nm, λ_em_ = 545 nm, Δ*t* = 10 sec).

**Figure S6.** (a) Time-dependent change in fluorescence spectra after treatment of βGlcNAc-CM-Rhod-P (10 μM) with dual-specificity phosphatase 15 (DUSP15, 100 nM) in 50 mM Tris buffer (pH 7.4) containing 1% DMSO (λ_ex_ = 510 nm, Δ*t* = 10 sec). (b) Time-dependent fluorescence response of βGlcNAc-CM-Rhod-P (10 μM) to DUSP15 (100 nM) in the absence or presence of 1 mM Na_3_VO_4_ (λ_ex_ = 510 nm, λ_em_ = 545 nm, Δ*t* = 10 sec).

**Figure S7.** Time-dependent change in absorption spectra after treatment of βGlcNAc-CM-Rhod-P (10 μM) with both OGA (100 nM) and ALP (100 nM) in 50 mM Tris buffer (pH 7.4) containing 1% DMSO.

**Figure S8.** (a) Fluorescence response of βGlcNAc-CM-Rhod-P (10 μM) upon titration with OGA (10-100 nM) (λ_ex_ = 400 nm, ΔF_450 nm_ = F_1_ – F_0_, F_0_: fluorescence intensity of untreated βGlcNAc-CM-Rhod-P at 450 nm, F_1_: fluorescence intensity arising from the probe at 450 nm after treatment with the indicated concentration of OGA. (b) Fluorescence response of βGlcNAc-CM-Rhod-P (10 μM) upon titration with ALP (10-100 nM) (λ_ex_ = 510 nm, ΔF_545 nm_ = F_1_ – F_0_, F_0_: fluorescence intensity of untreated βGlcNAc-CM-Rhod-P at 545 nm, F_1_: fluorescence intensity arising from the probe at 545 nm after treatment with the indicated concentration of ALP).

**Figure S9.** Reversed-phase HPLC analysis of products obtained by treatment of 10 μM βGlcNAc-CM-Rhod-P with OGA (100 nM) or/and ALP (100 nM) in the absence and presence of 50 μM PUGNAc or/and 1 mM Na_3_VO_4_ (**a** = βGlcNAc-CM-Rhod-P ([M + H]^+^: m/z = 872.4), **b** = βGlcNAc-CM-Rhod ([M + H]^+^: m/z = 792.2), **c** = CM-Rhod-P ([M + H]^+^: m/z = 669.1), **d** = CM-Rhod ([M + H]^+^: m/z = 589.1)).

**Figure S10.** Indicated cells were incubated with various concentrations of βGlcNac-CM-Rhod-P for 24 h. Cell death was determined by means of a MTT assay (mean ± s.d., n = 3).

**Figure S11.** AGS cells were incubated with various concentrations of μM βGlcNAc-CM-Rhod-P for 18 h. Cell images were obtained by using confocal fluorescence microscopy (scale bar = 5 μm). Graphs show the fluorescence intensity of Rhod (λ_ex_ = 488 nm) and CM (λ_ex_ = 405 nm) in cells (mean ± s.d., n = 3).

**Figure S12.** AGS cells were incubated with 100 μM βGlcNAc-CM-Rhod-P for indicated time periods. Cell images were obtained by using confocal fluorescence microscopy (scale bar = 5 μm). Graphs show the fluorescence intensity of Rhod (λ_ex_ = 488 nm) and CM (λ_ex_ = 405 nm) in cells (mean ± s.d., n = 3).

**Figure S13.** Graphs show the fluorescence intensity of CM and Rhod after incubation of βGlcNAc-CM-Rhod-P with lysates of the indicated cells (Un = untreated with the probe) (mean ± s.d., n = 3).

**6. NMR spectra**

**Compound 2 (DMSO-*d*_6_, 400 MHz ^1^H NMR, 100 MHz ^13^C NMR)**


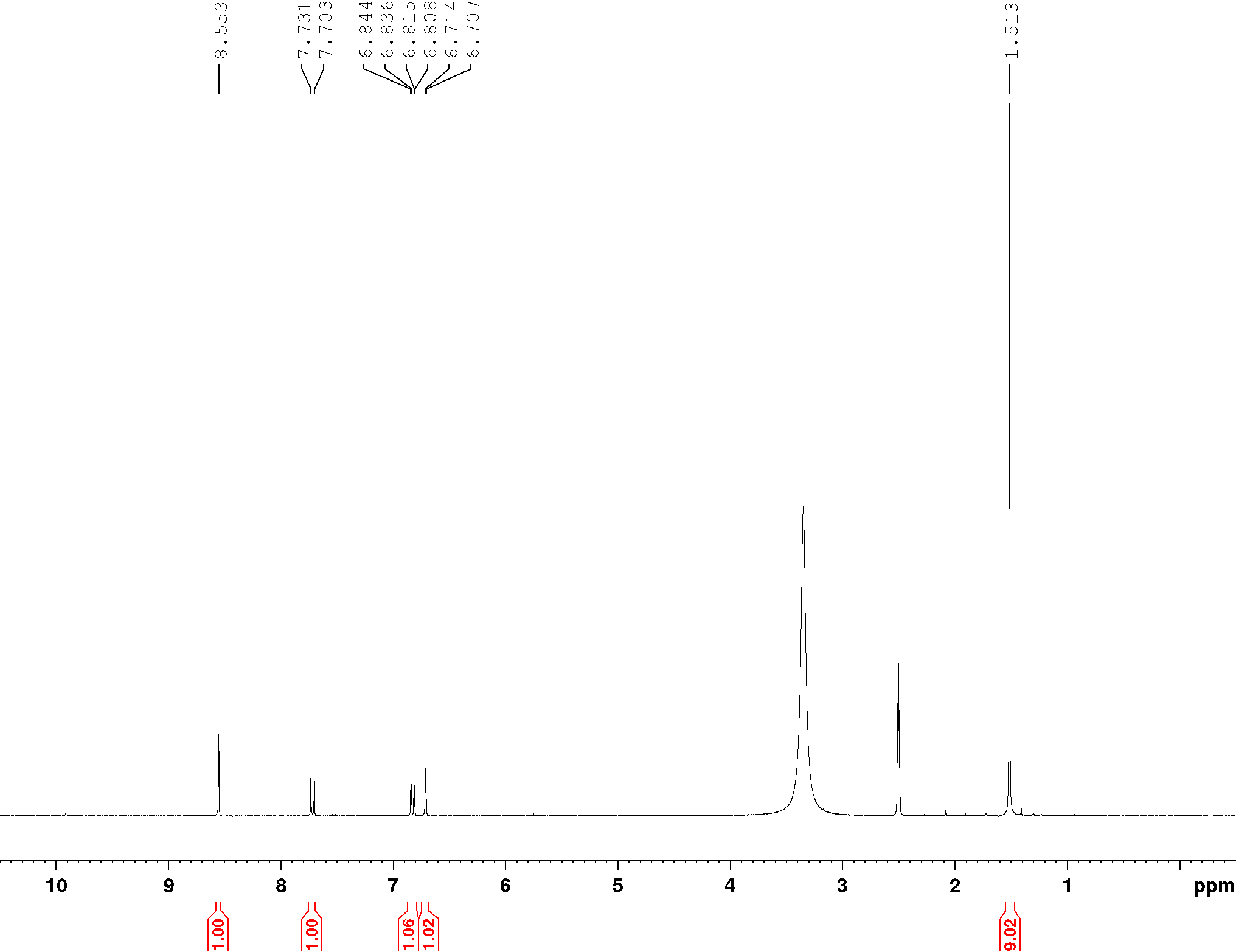


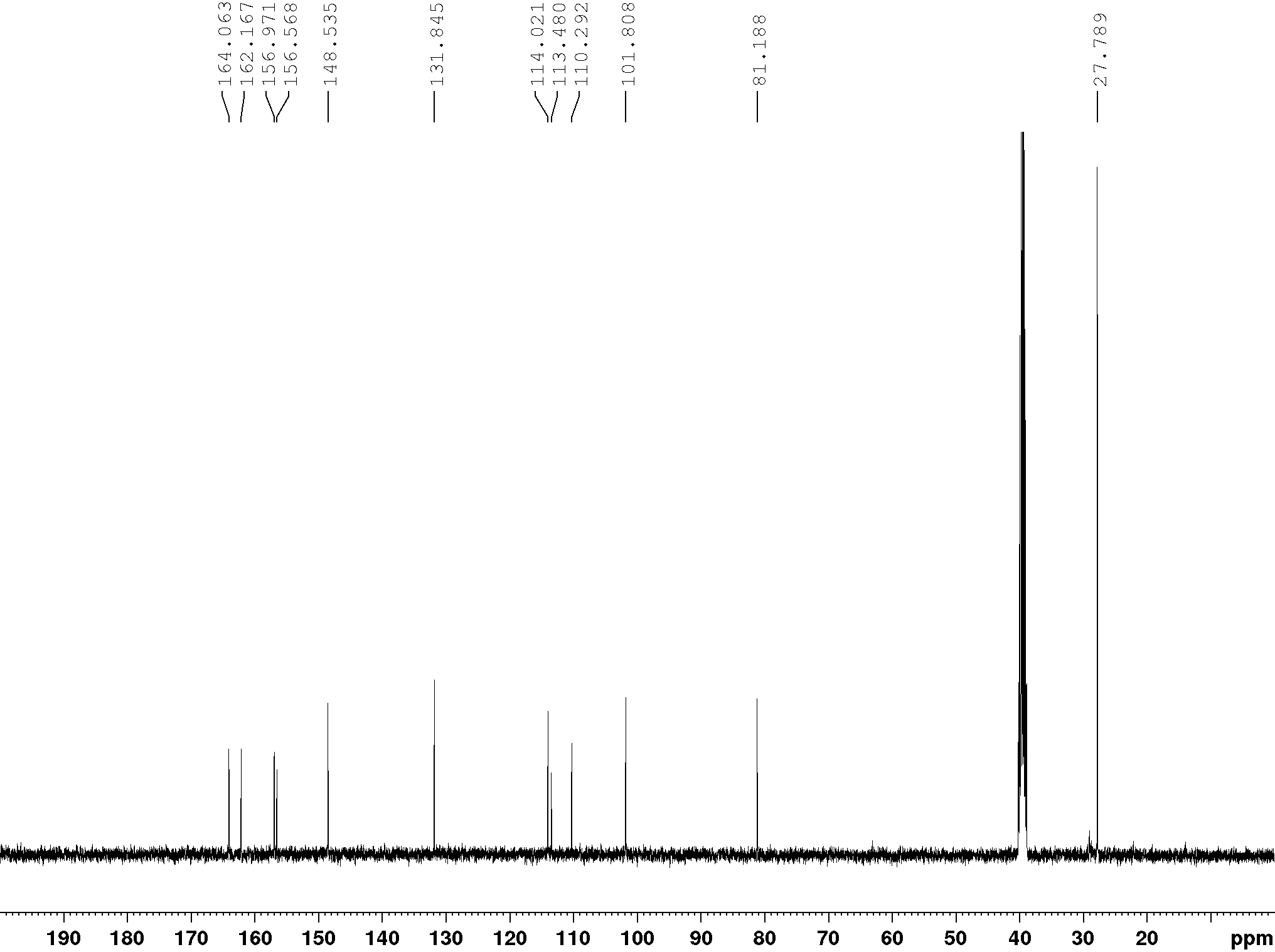


**Compound 3 (CDCl_3_, 400 MHz ^1^H NMR, 100 MHz ^13^C NMR)**


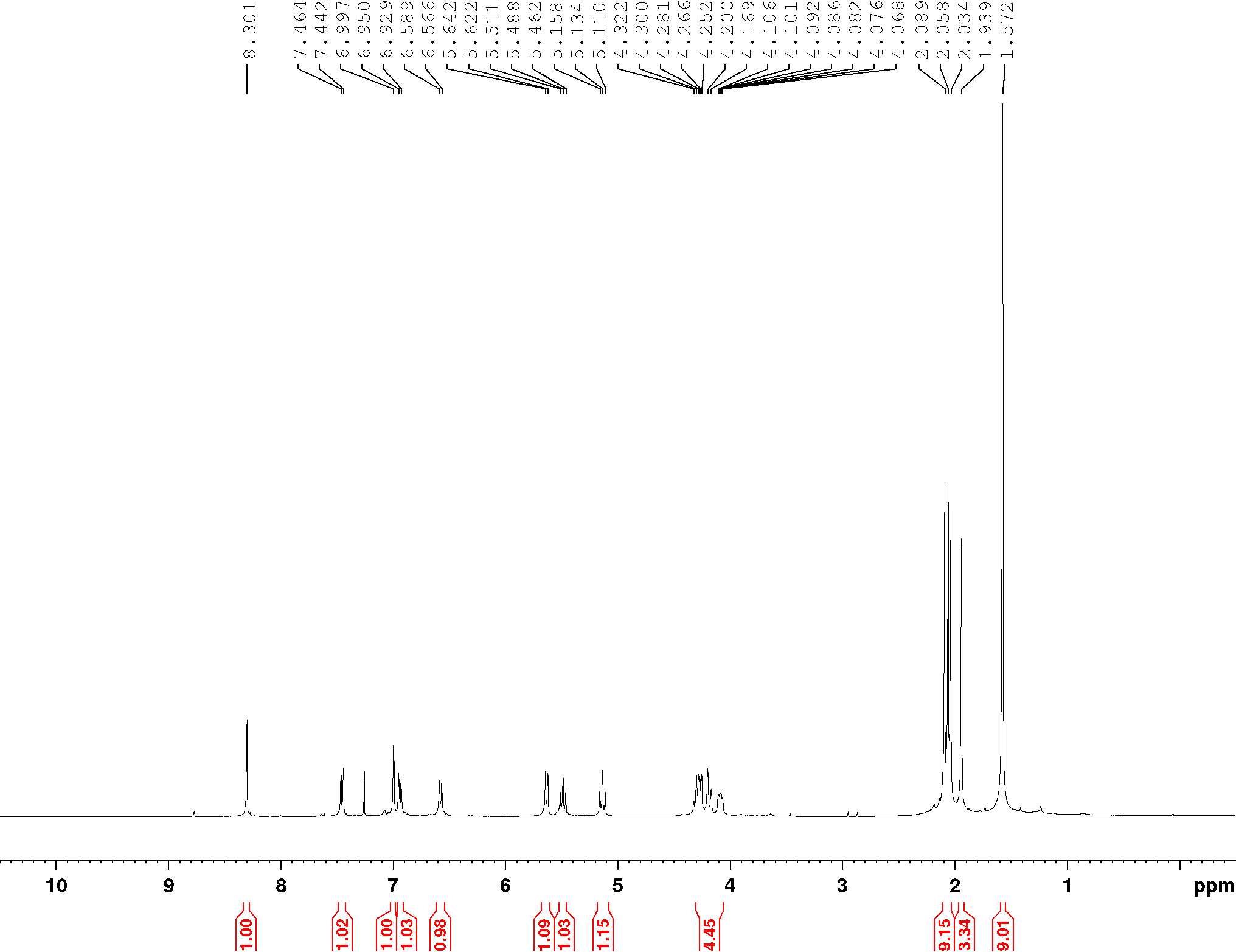


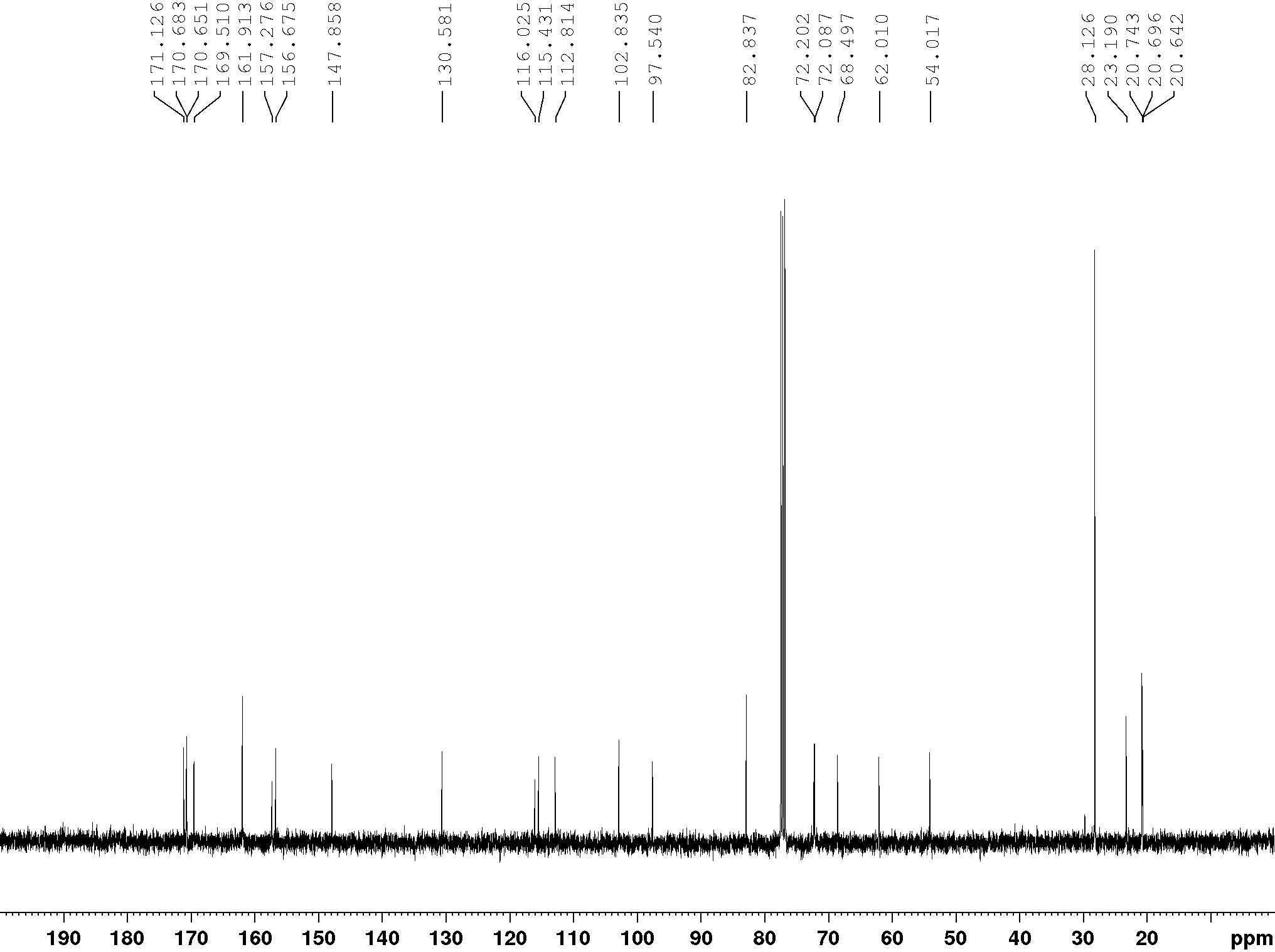


**Compound 4 (DMSO-*d*_6_, 400 MHz ^1^H NMR, 100 MHz ^13^C NMR)**


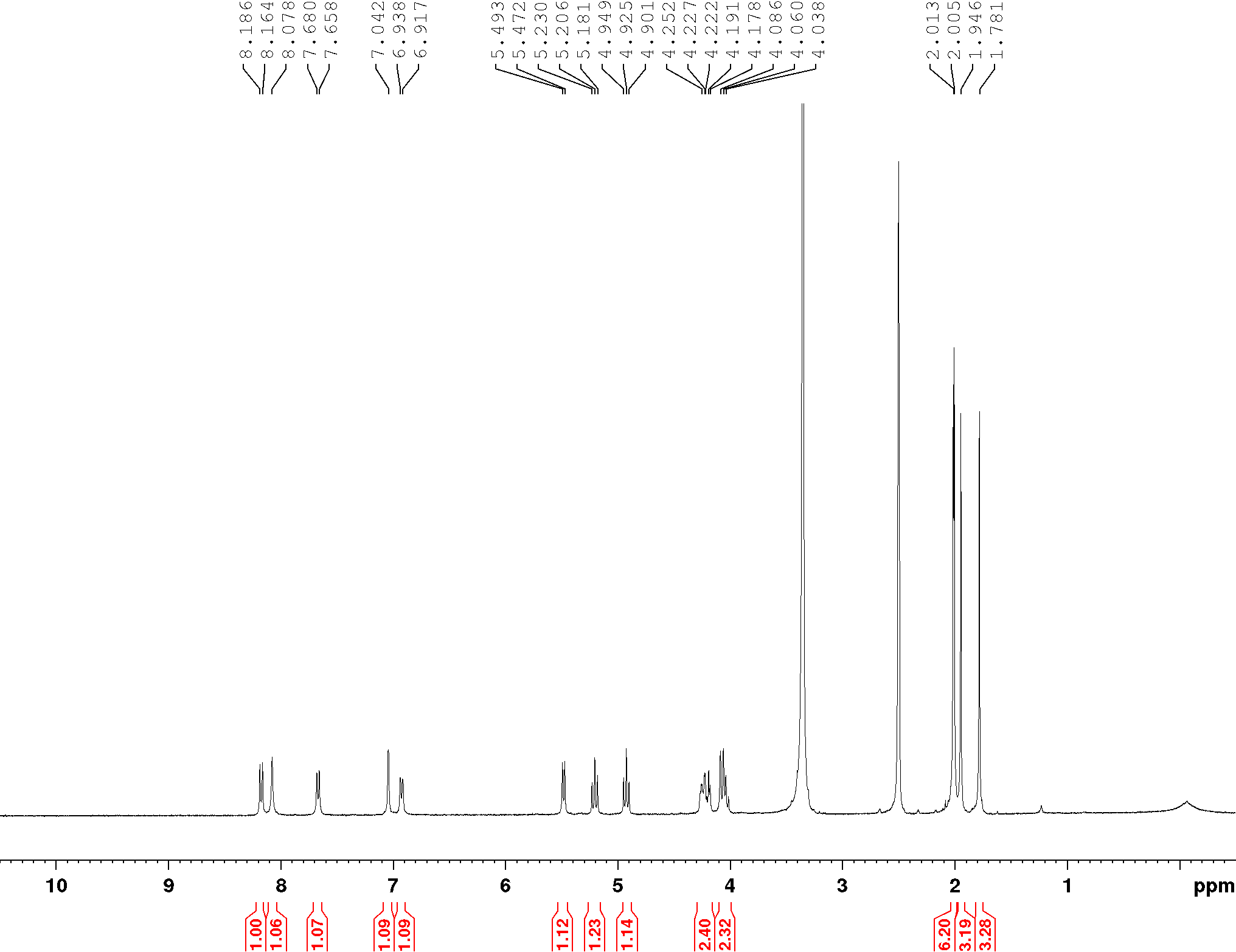


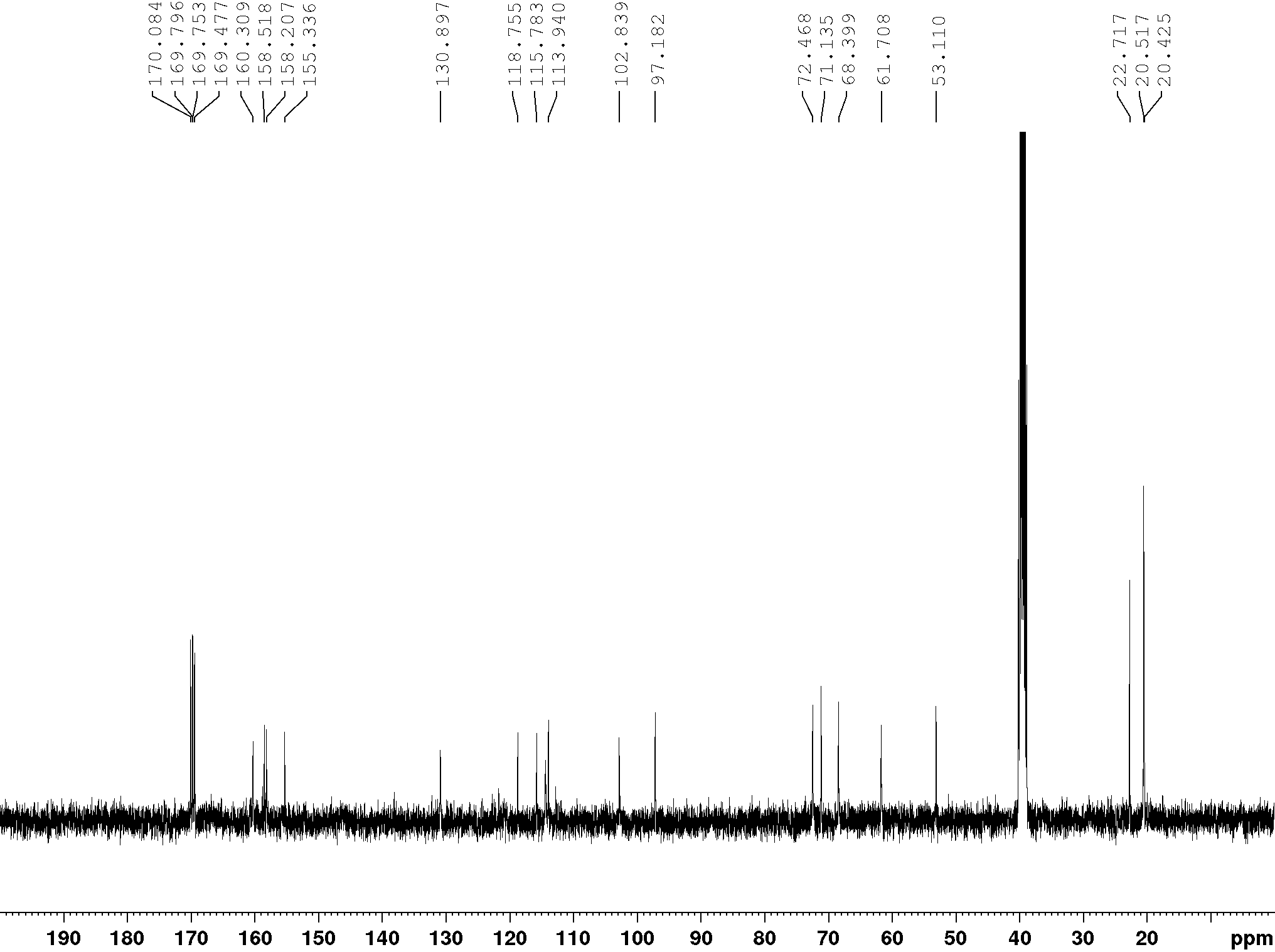


**Compound 5 (DMSO-*d*_6_, 400 MHz ^1^H NMR, 100 MHz ^13^C NMR)**

**
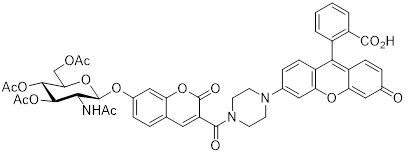
**
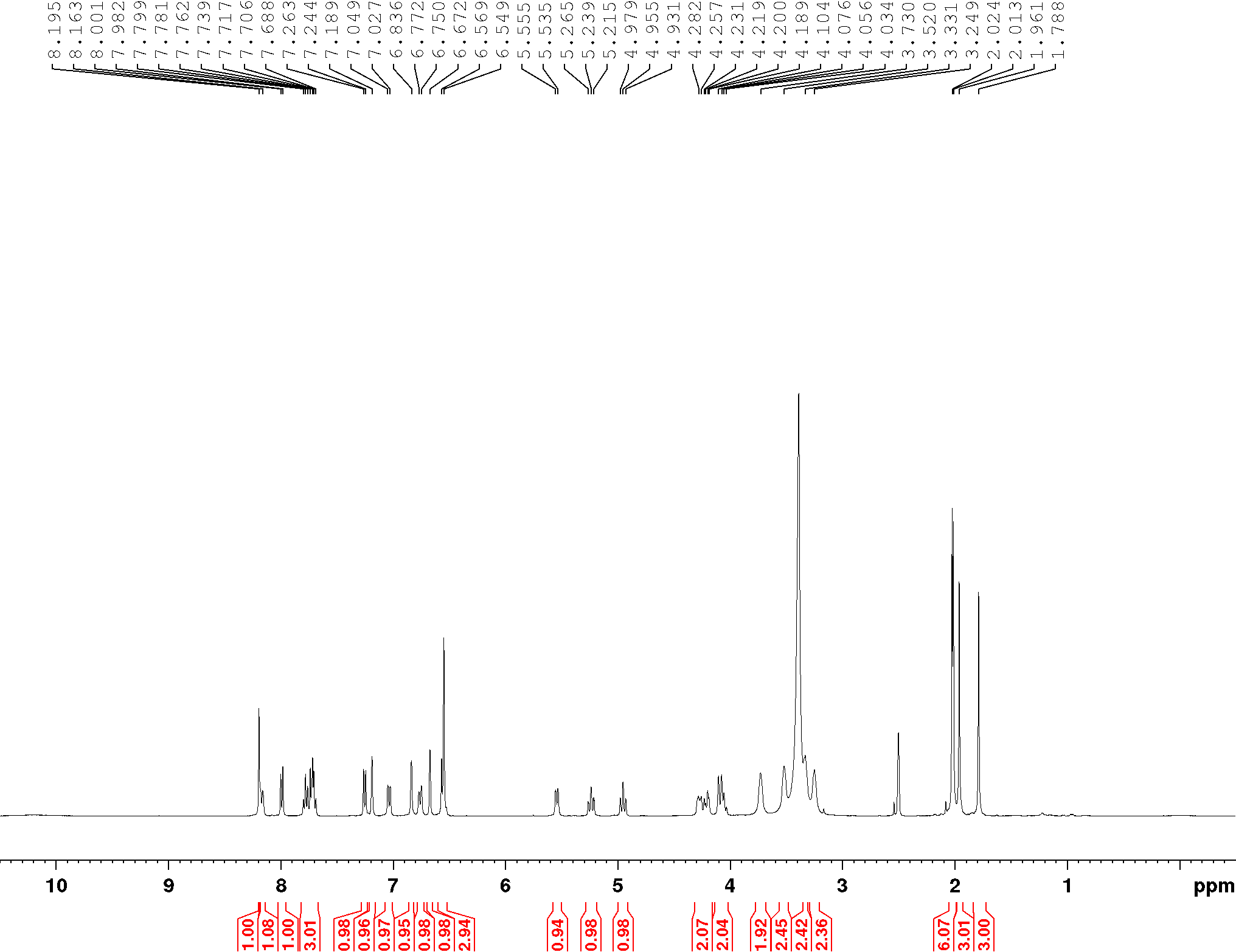


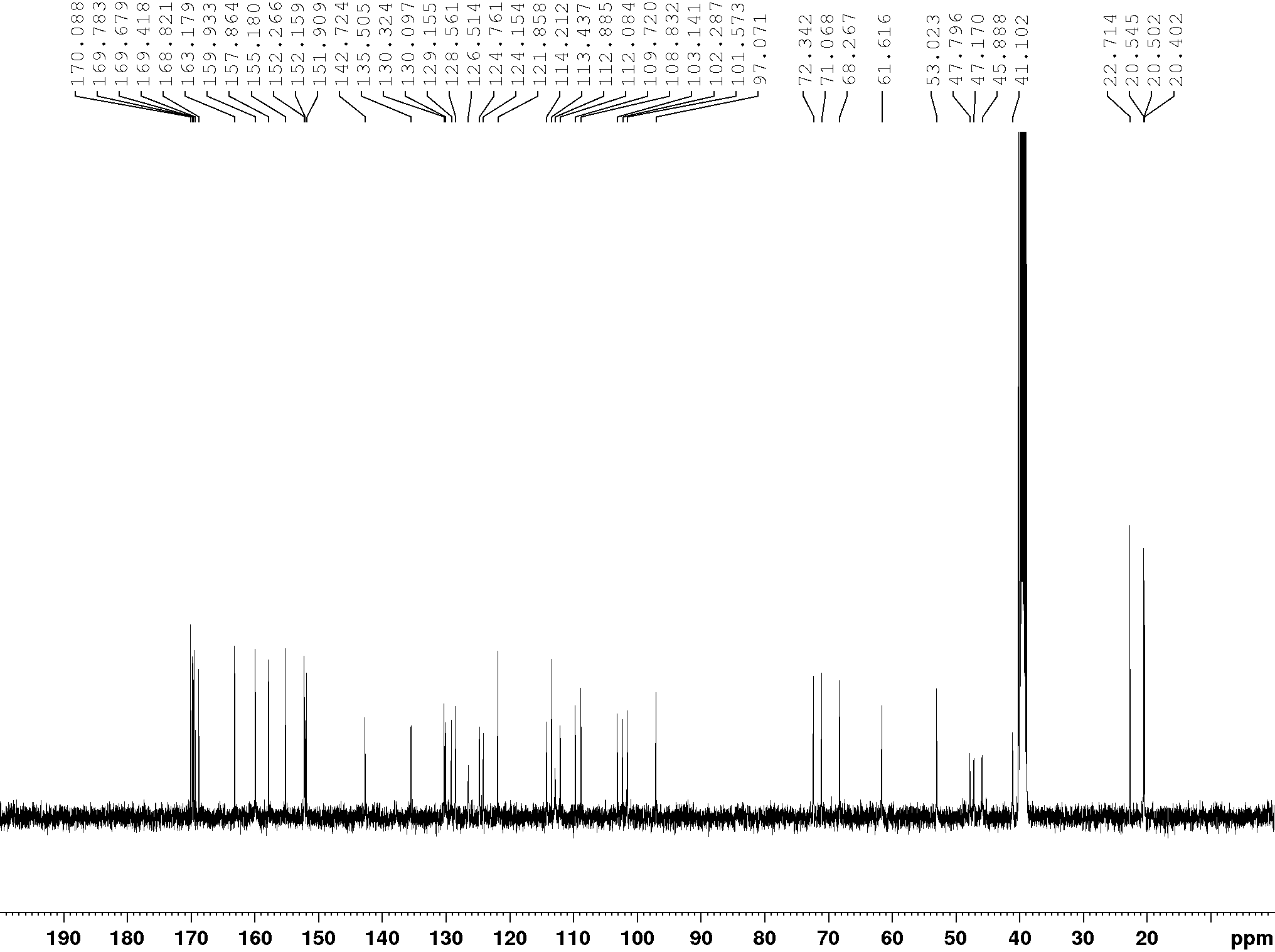


**Compound 6 (DMSO-*d*_6_, 400 MHz ^1^H NMR, CD_3_OD, 100 MHz ^13^C NMR, 162 MHz ^31^P NMR)**


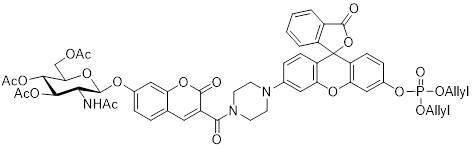

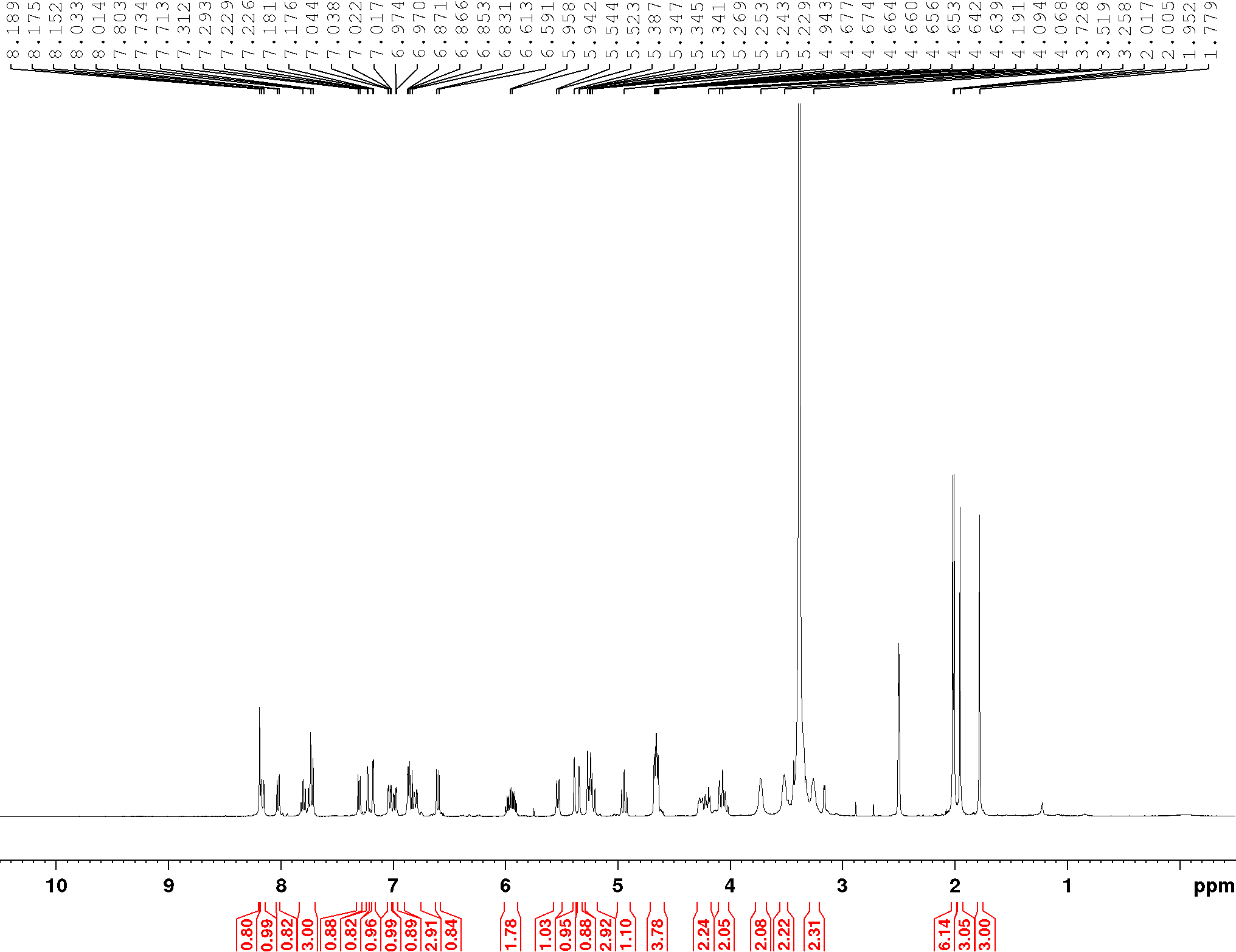


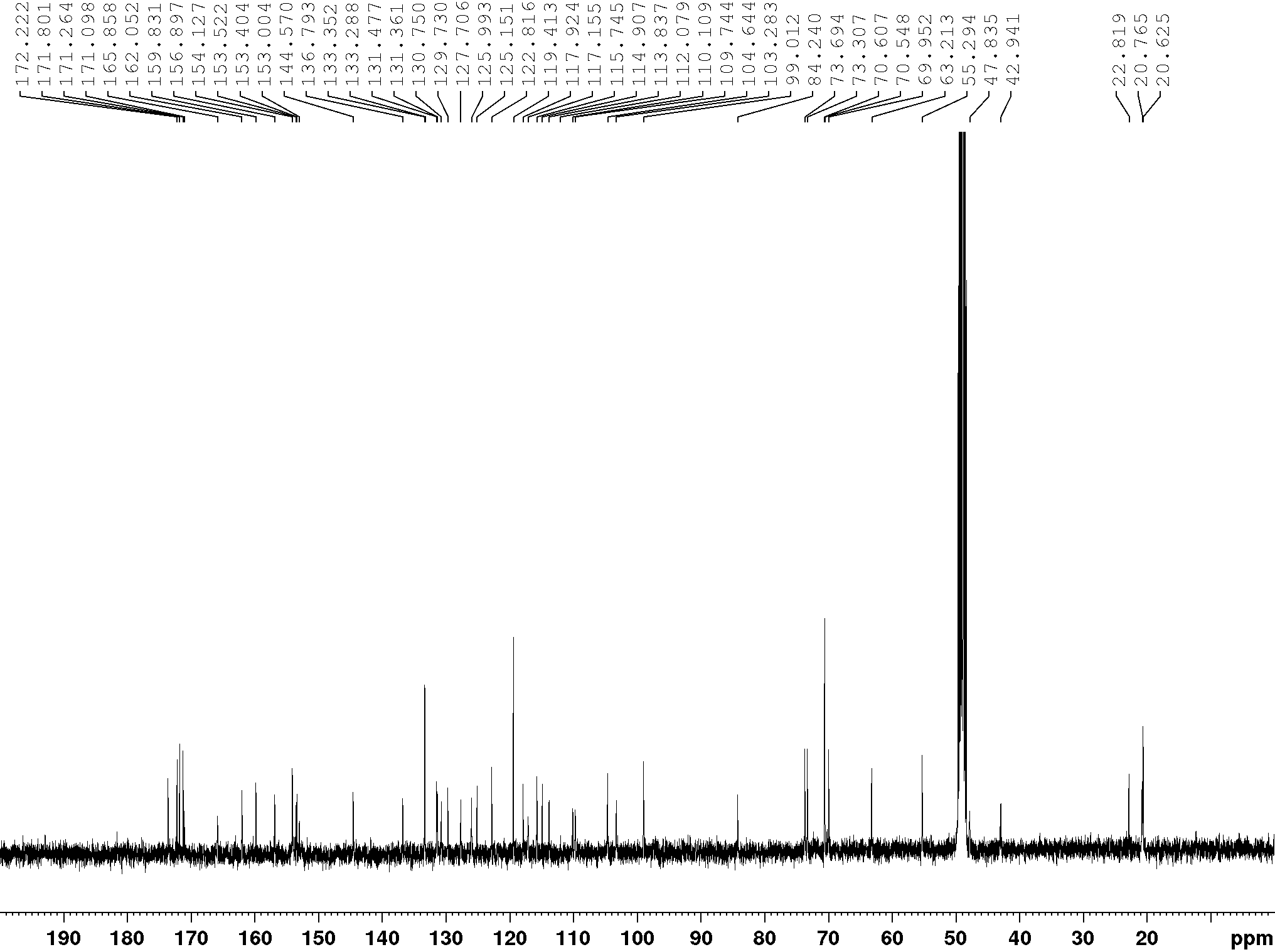


**βGlcNAc-CM-Rhod-P (DMSO-*d*_6_, 400 MHz ^1^H NMR, 100 MHz ^13^C NMR, 162 MHz ^31^P NMR)**


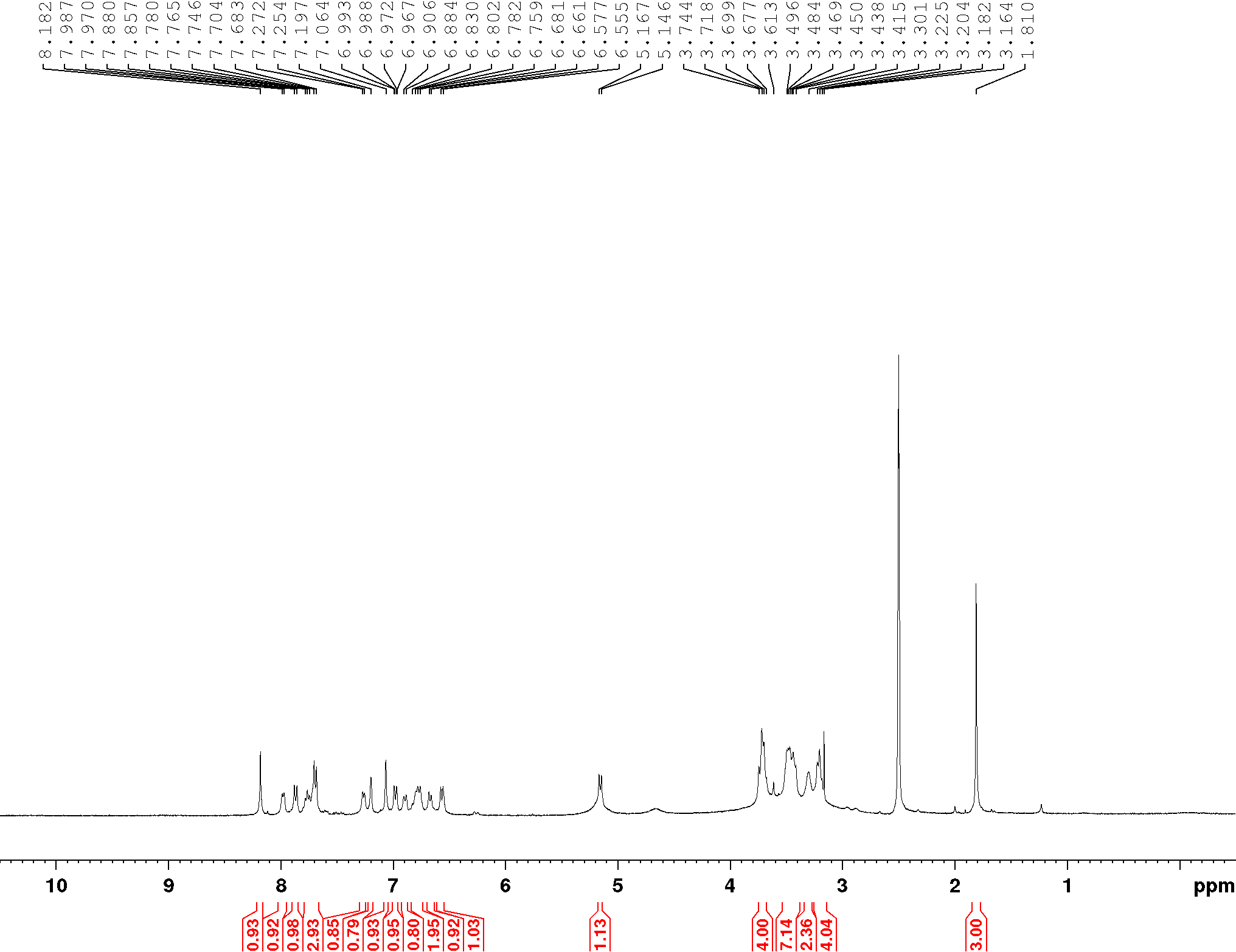


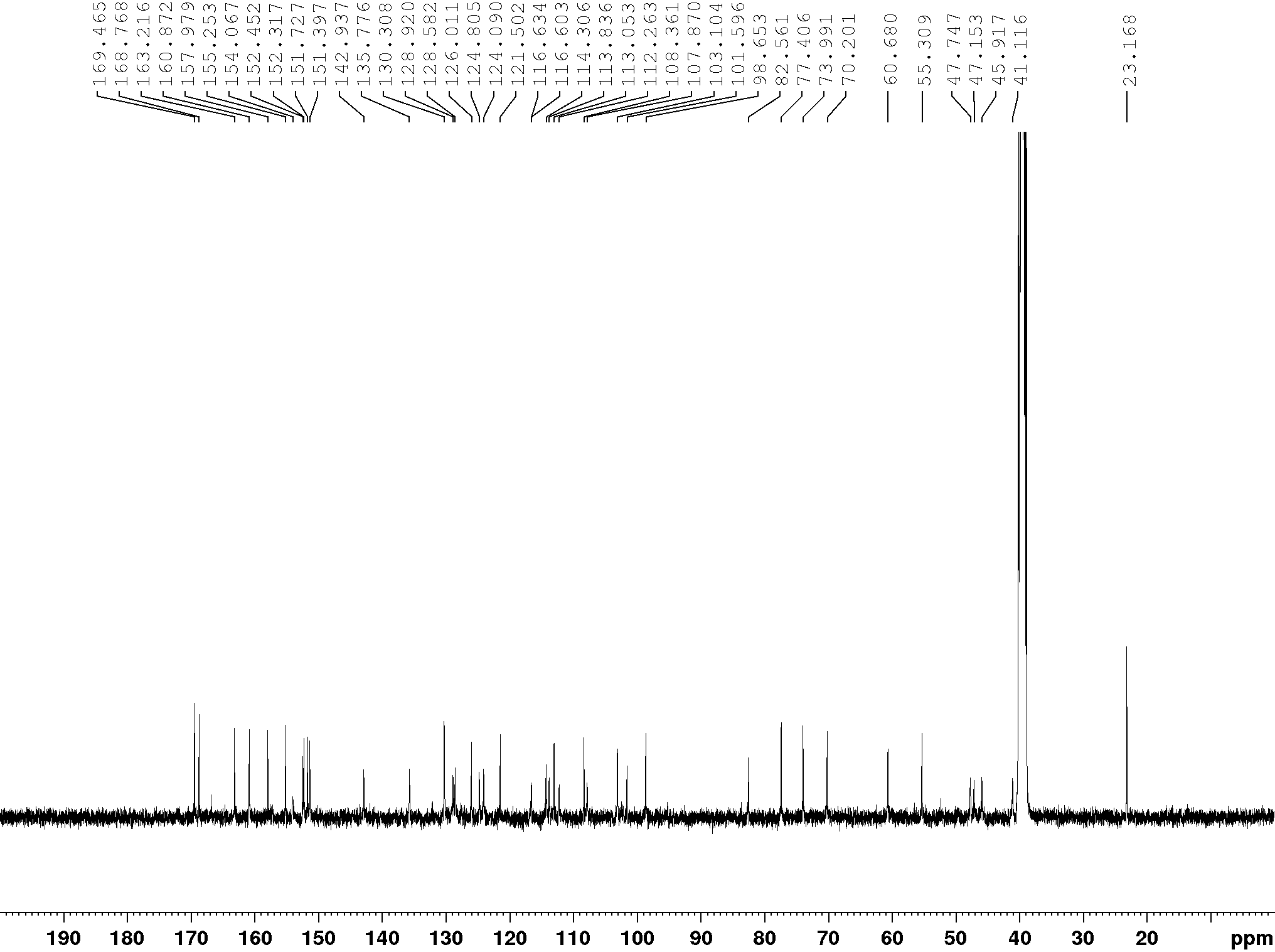


**High-resolution mass spectrum of βGlcNAc-CM-Rhod-P**

**CM-Rhod (DMSO-*d*_6_, 400 MHz ^1^H NMR, 100 MHz ^13^C NMR)**


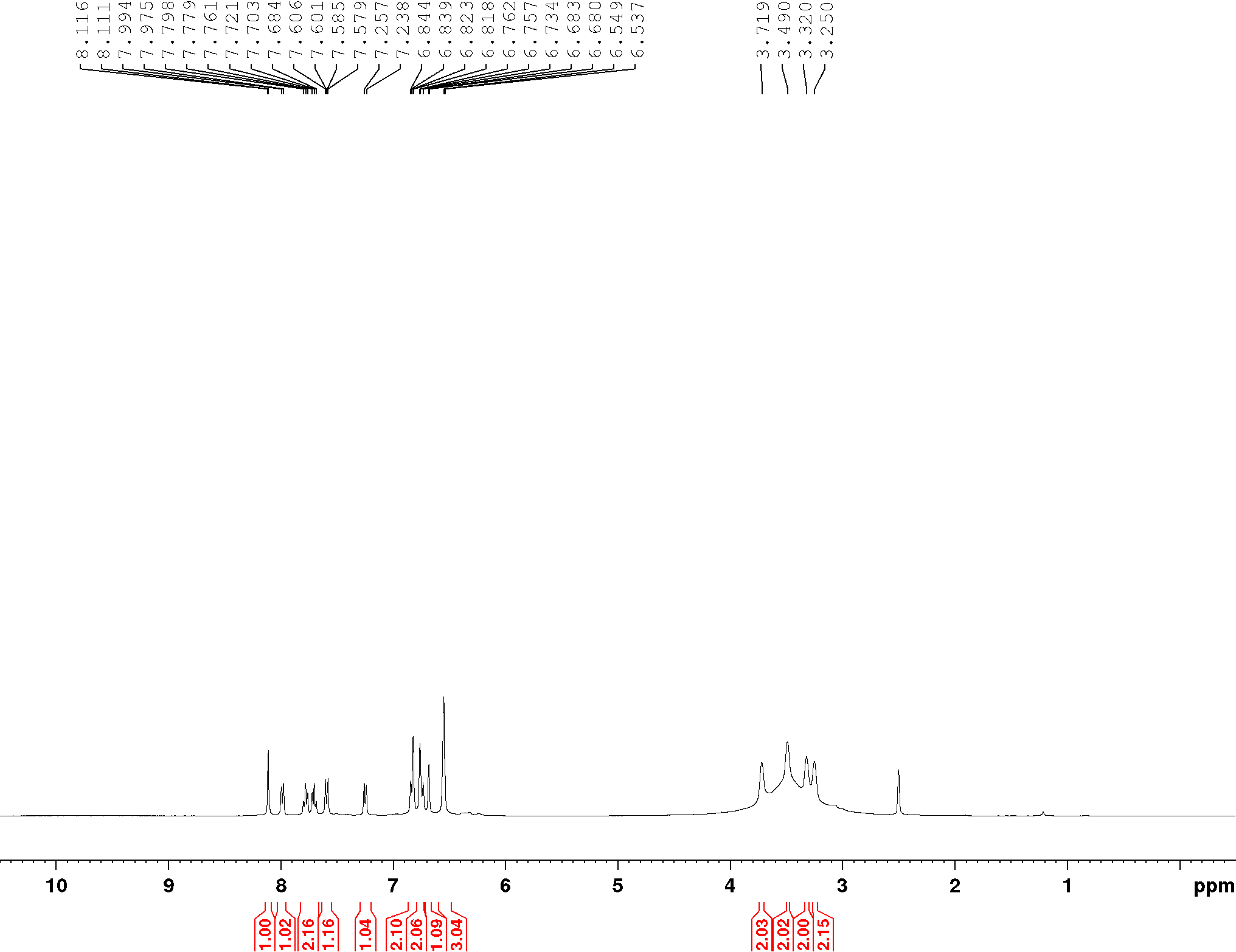


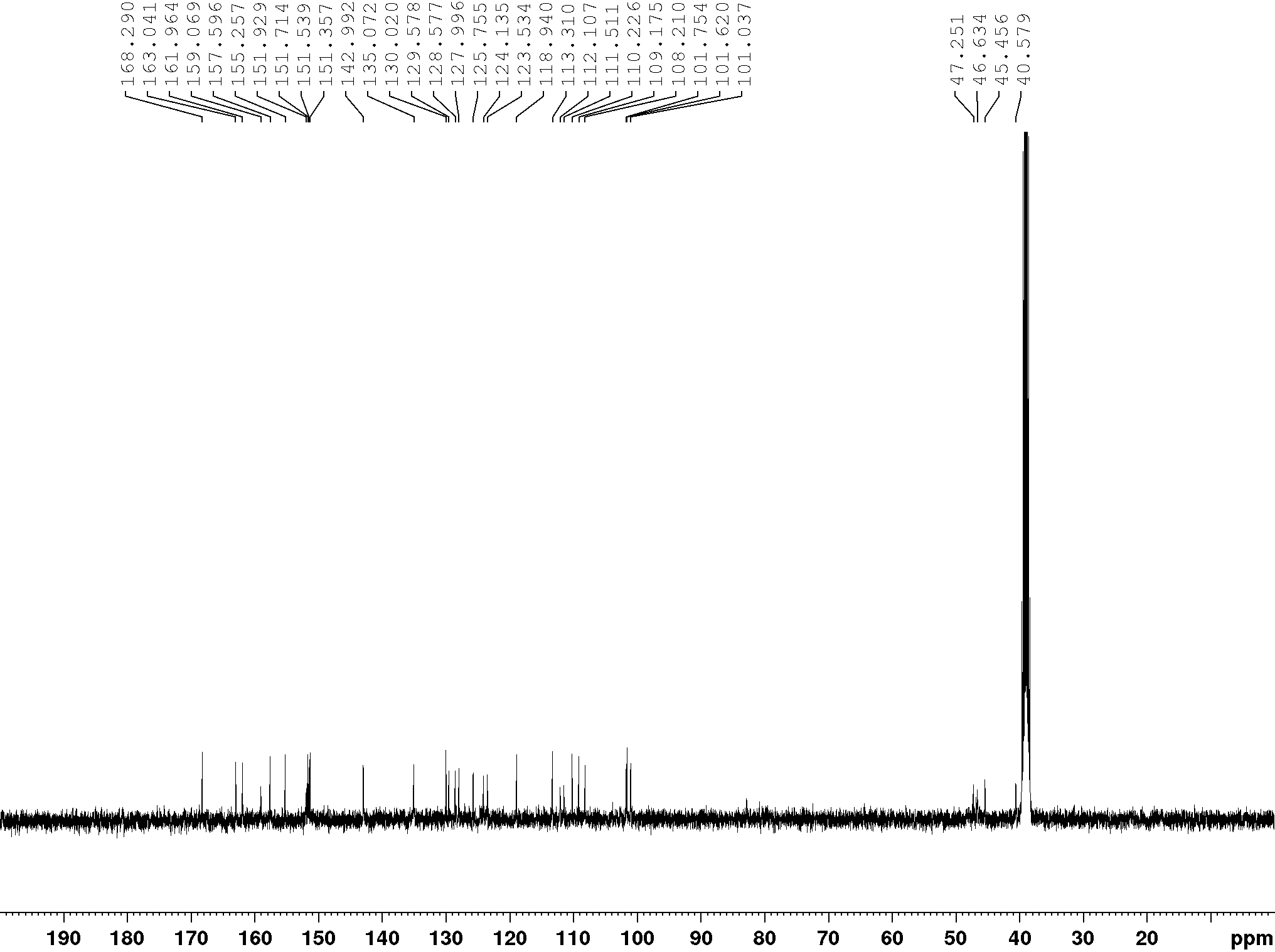


**βGlcNAc-CM-Rhod (CD_3_OD, 400 MHz ^1^H NMR, DMSO-*d*_6_, 100 MHz ^13^C NMR)**

**
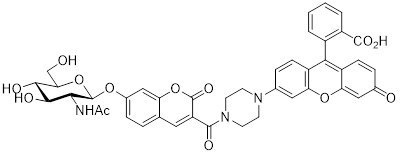
**
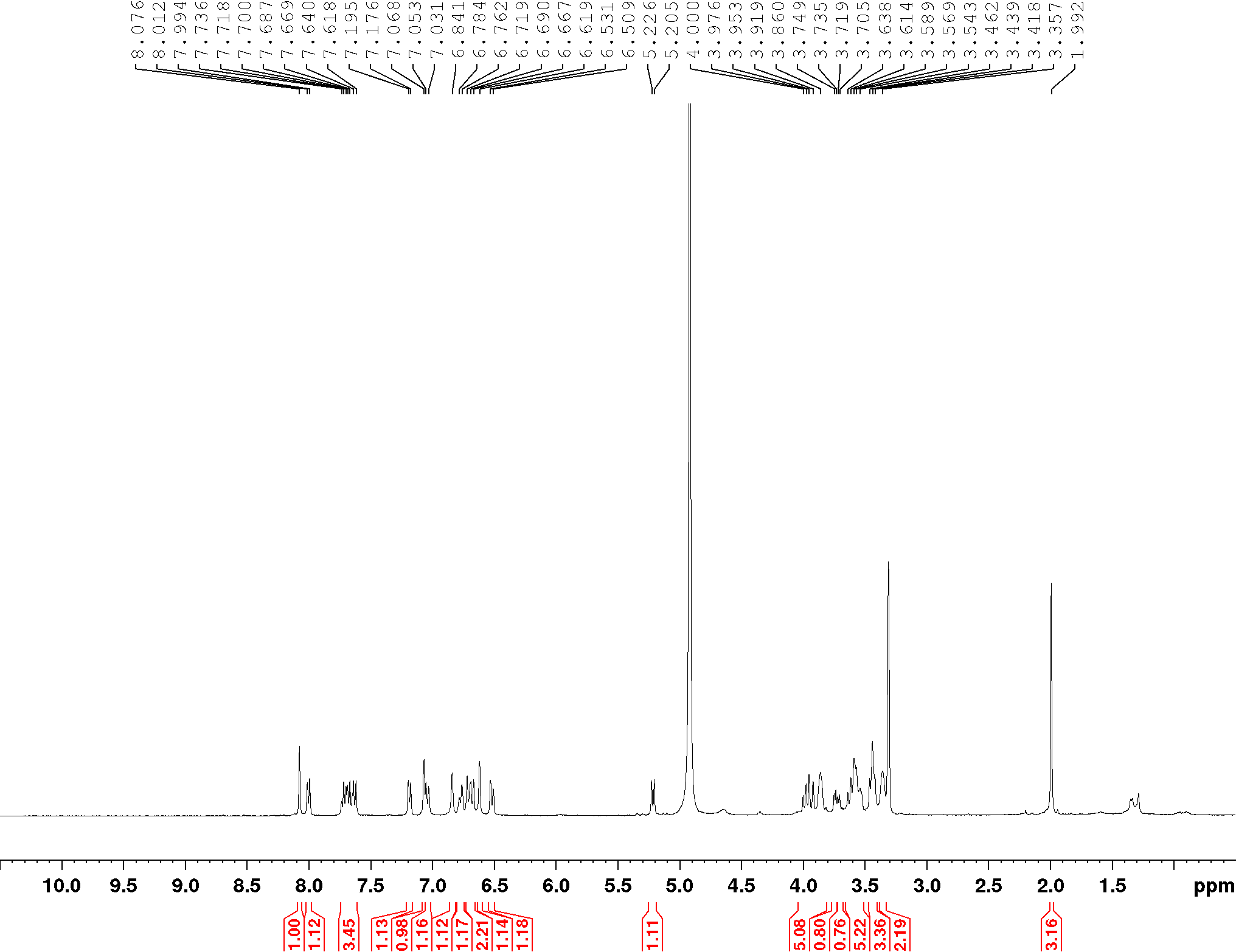


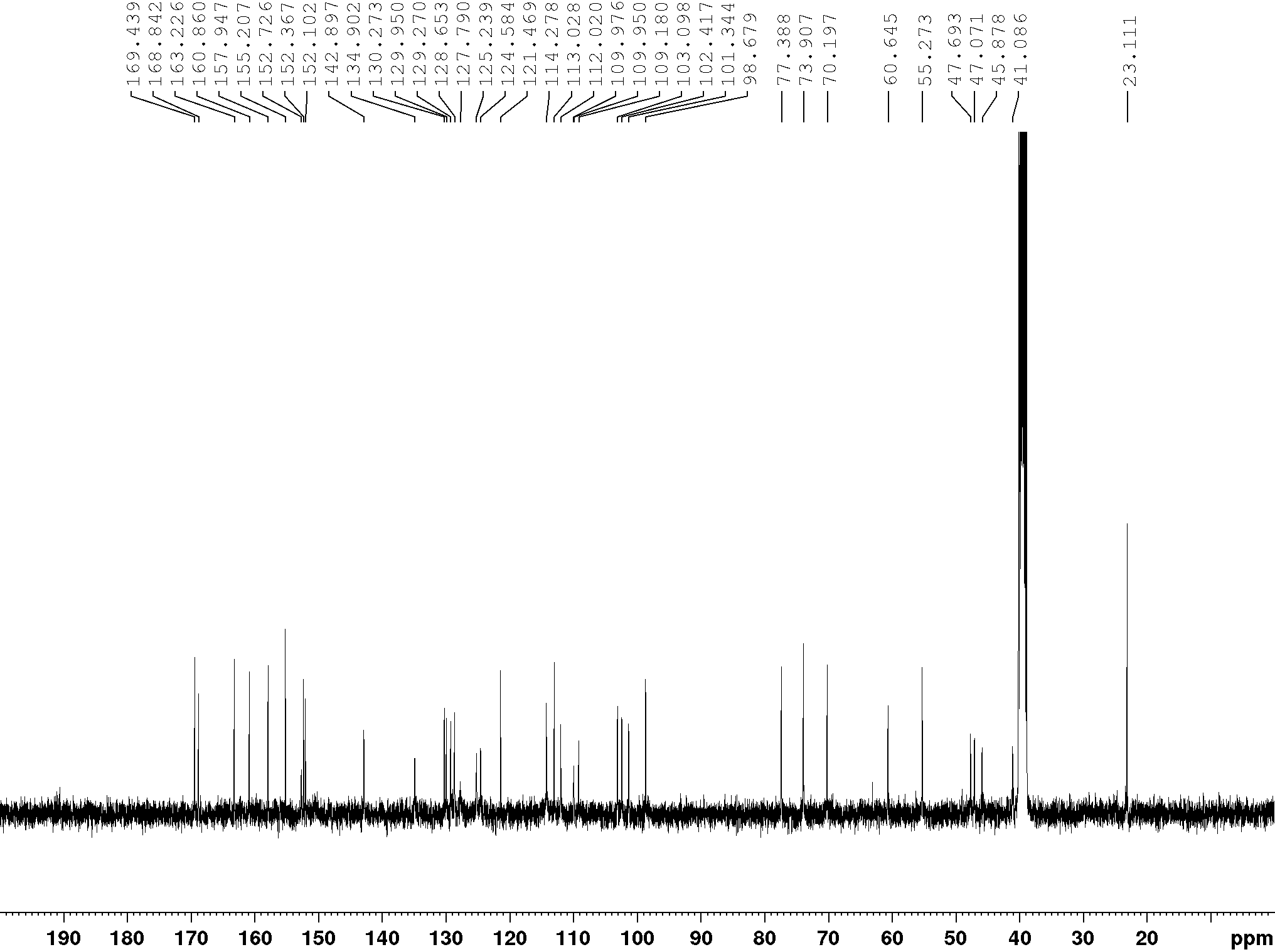


**Compound 8 (CDCl_3_, 400 MHz ^1^H NMR, 100 MHz ^13^C NMR)**


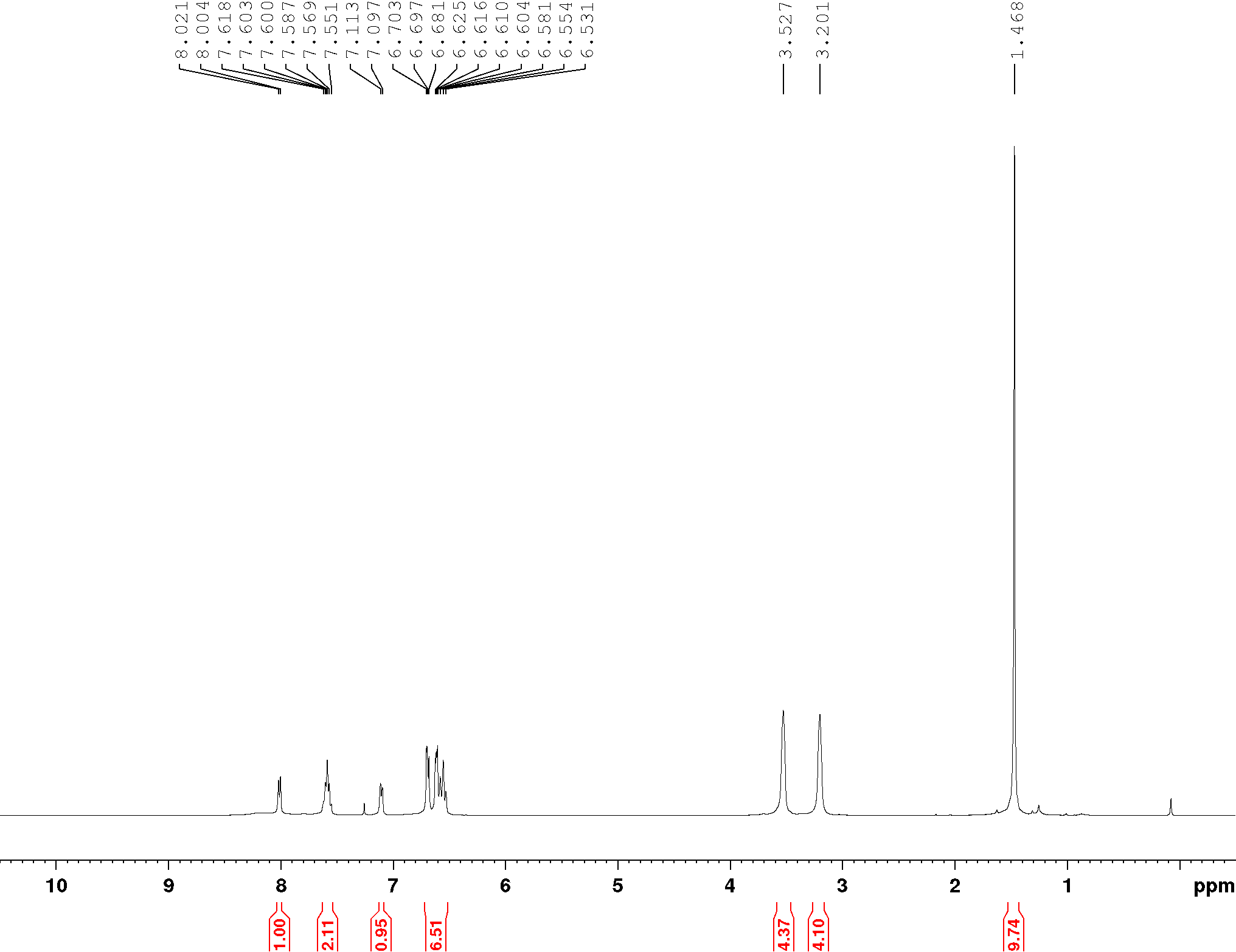


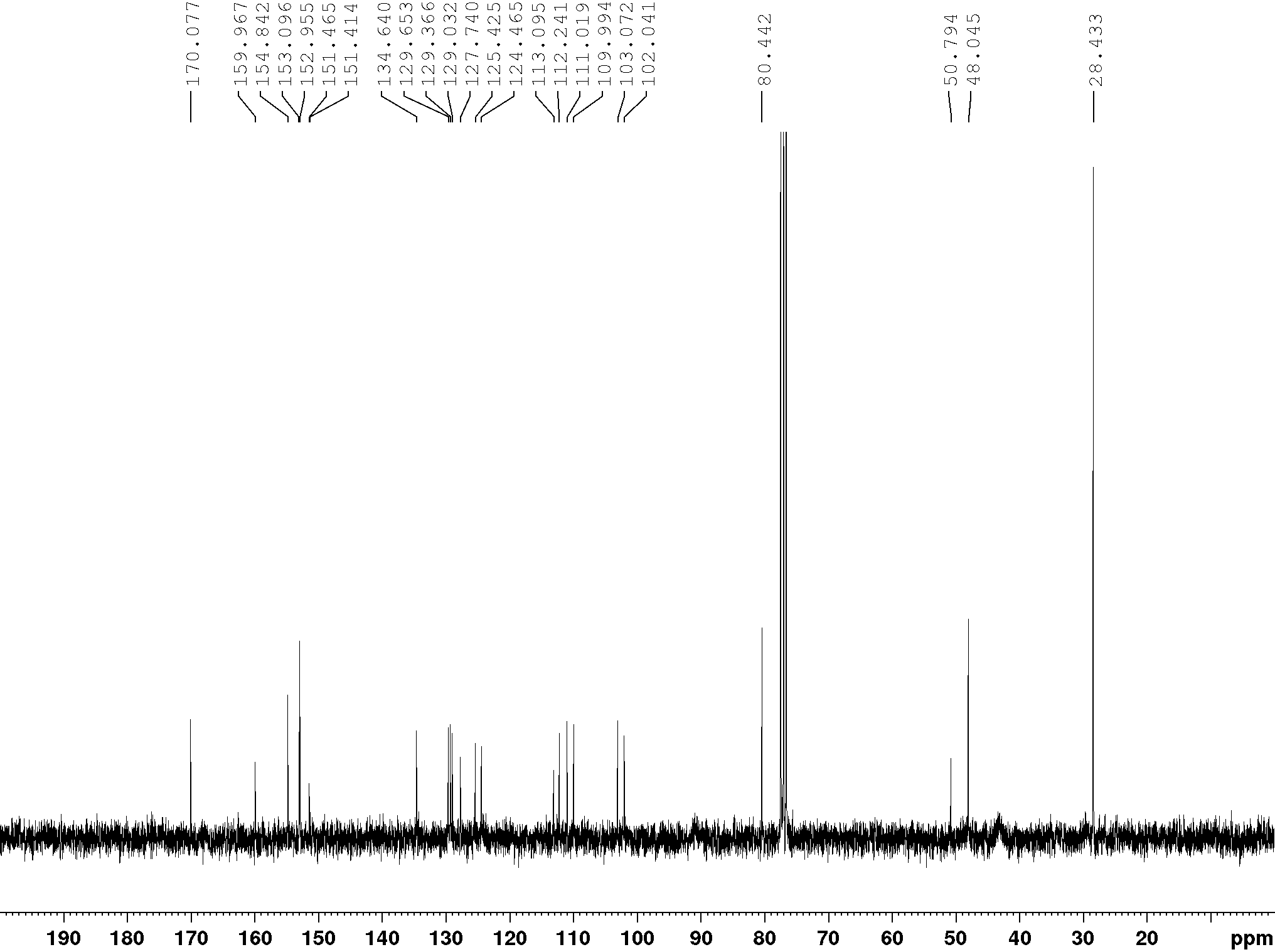


**Rhod (CDCl_3_, 400 MHz ^1^H NMR, 100 MHz ^13^C NMR)**

**
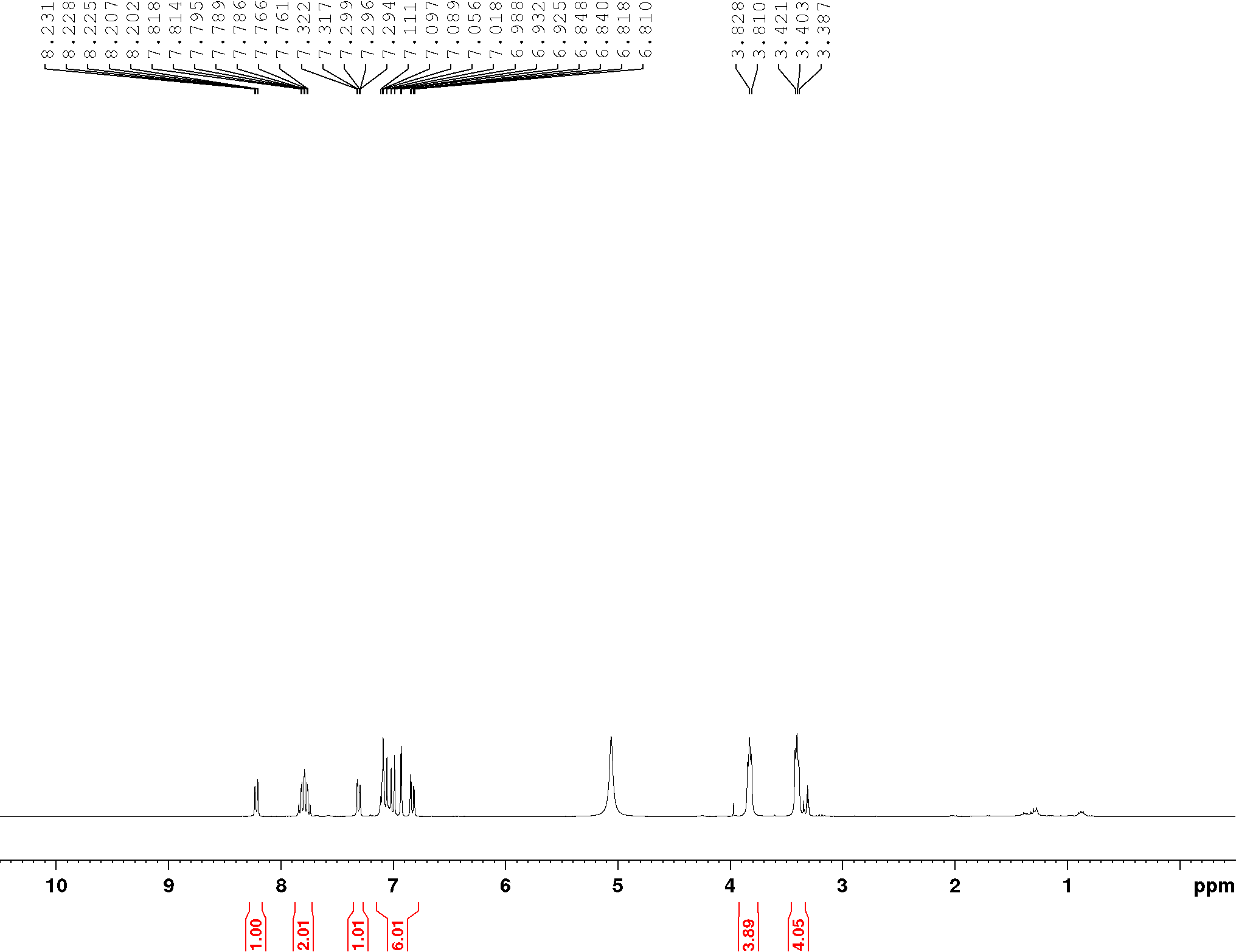
**

**
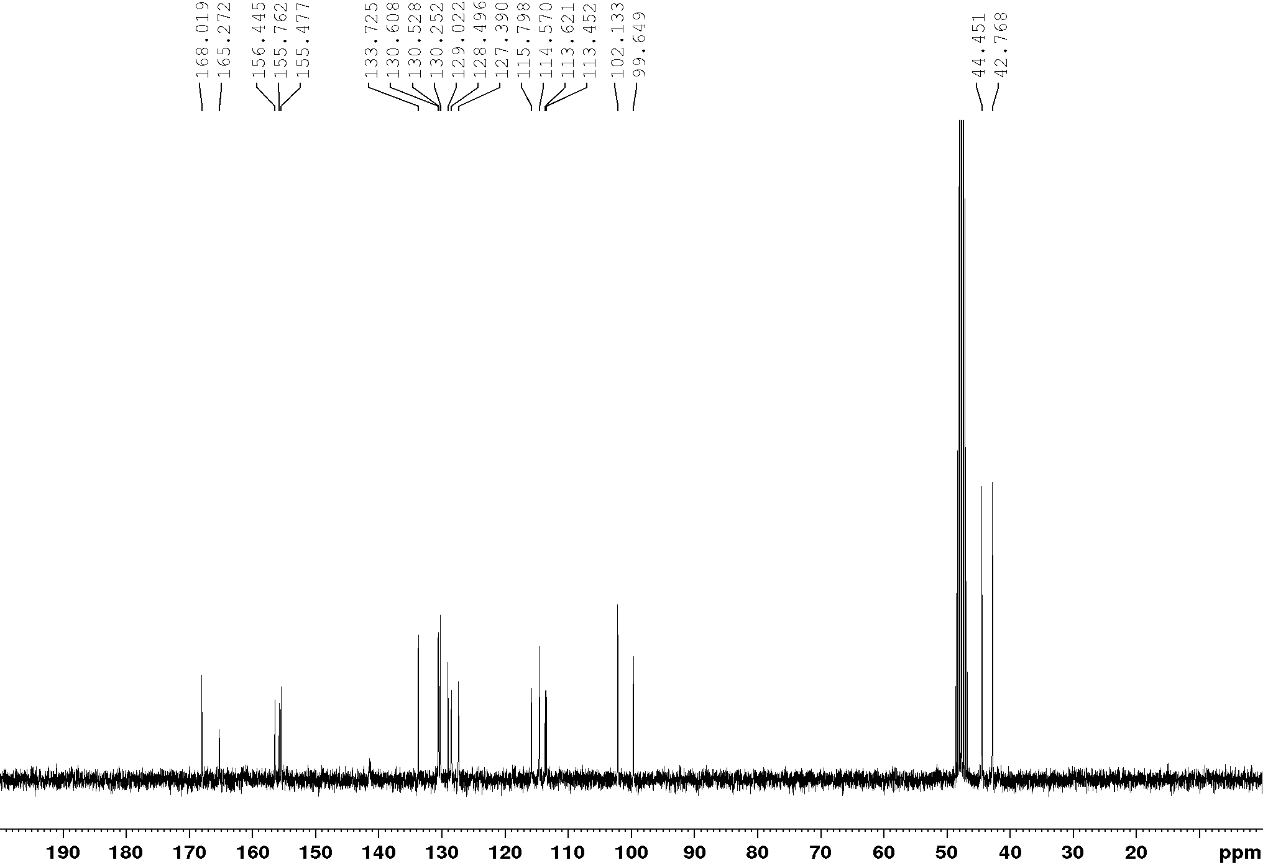
**

**Compound 9 (CDCl_3_, 400 MHz ^1^H NMR, 100 MHz ^13^C NMR, 162 MHz ^31^P NMR)**

**
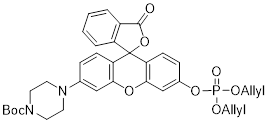

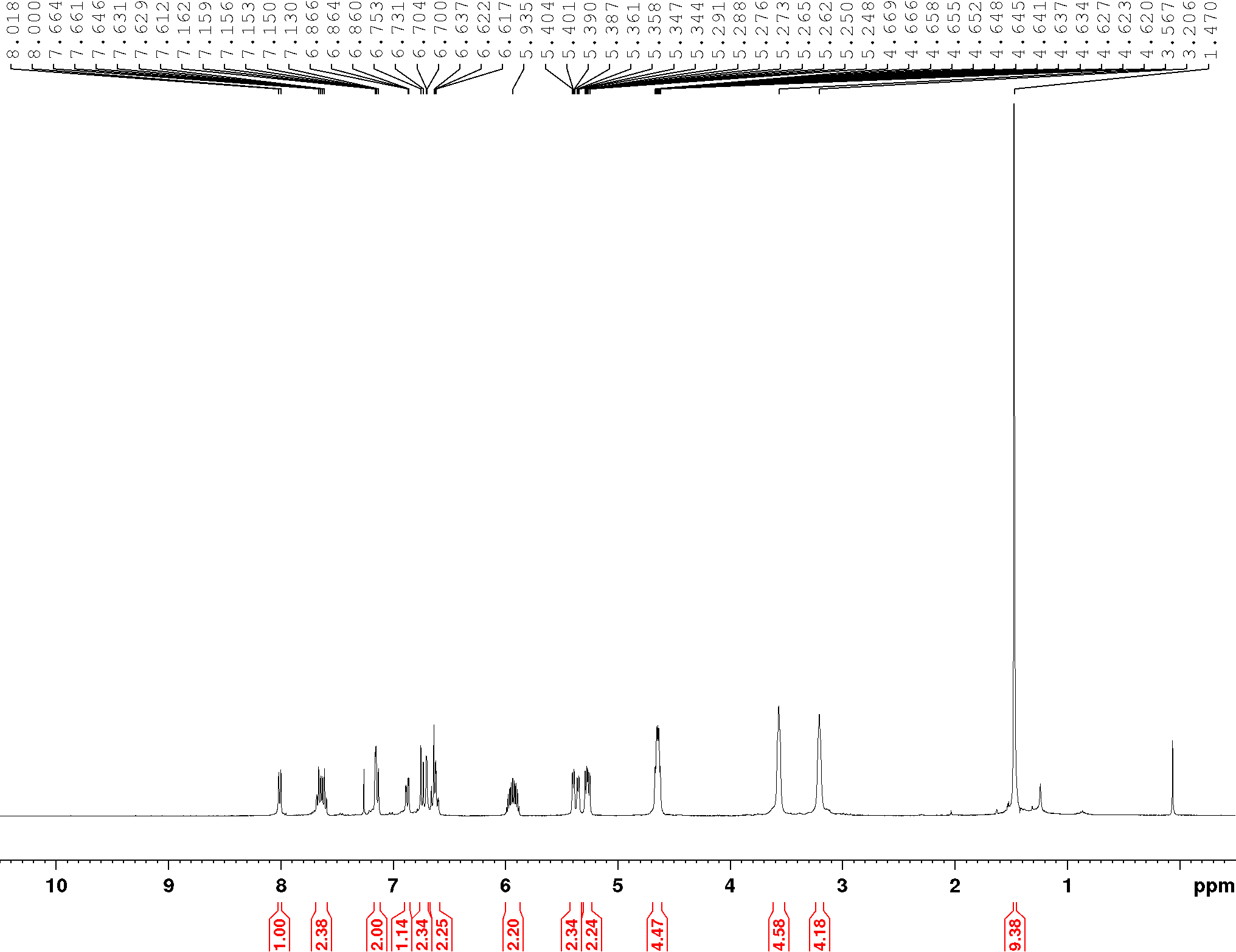
**


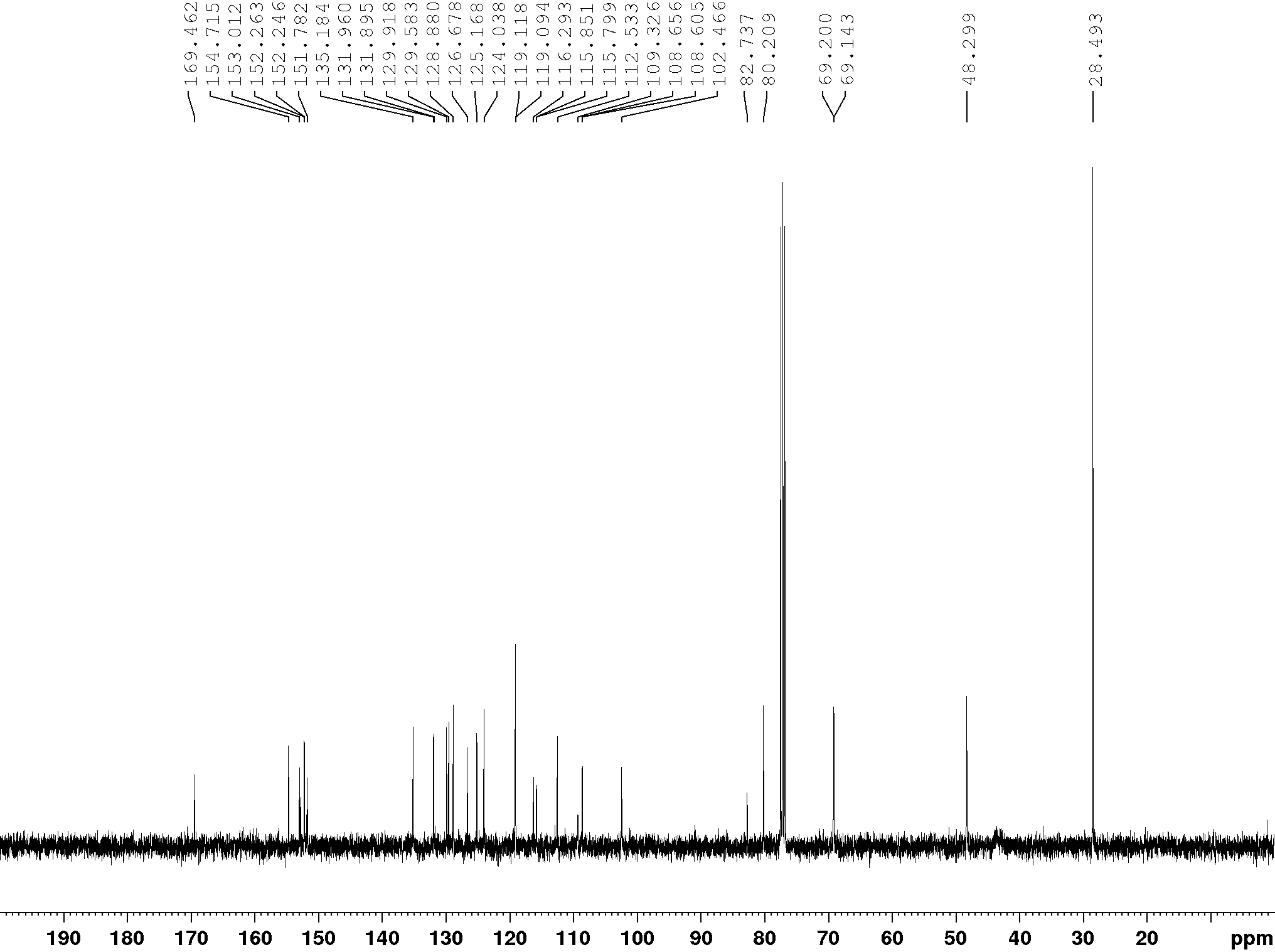


**Compound 10 (CD_3_OD, 400 MHz ^1^H NMR, 100 MHz ^13^C NMR, CDCl_3_, 162 MHz ^31^P NMR)**

**
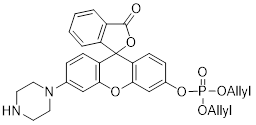
**
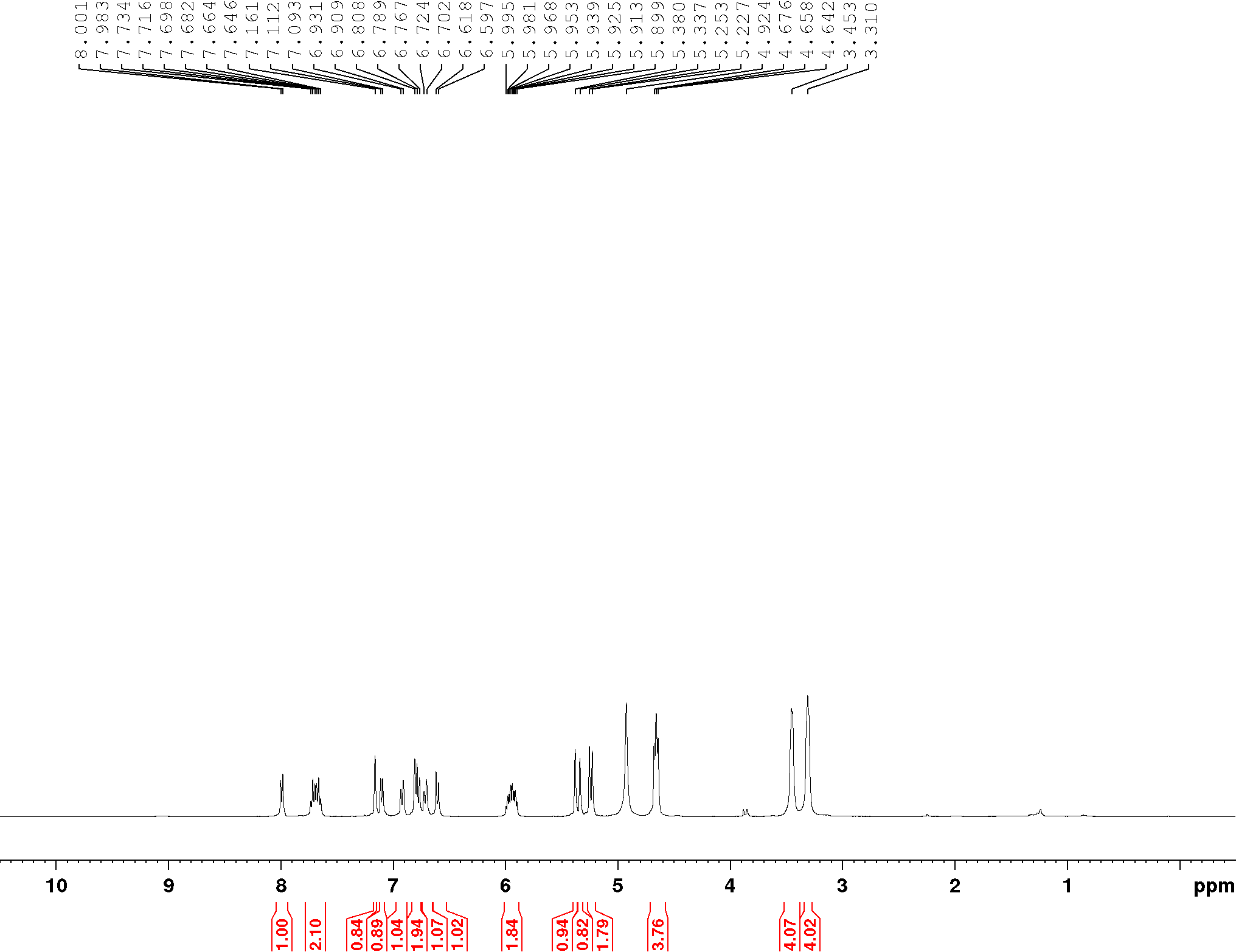


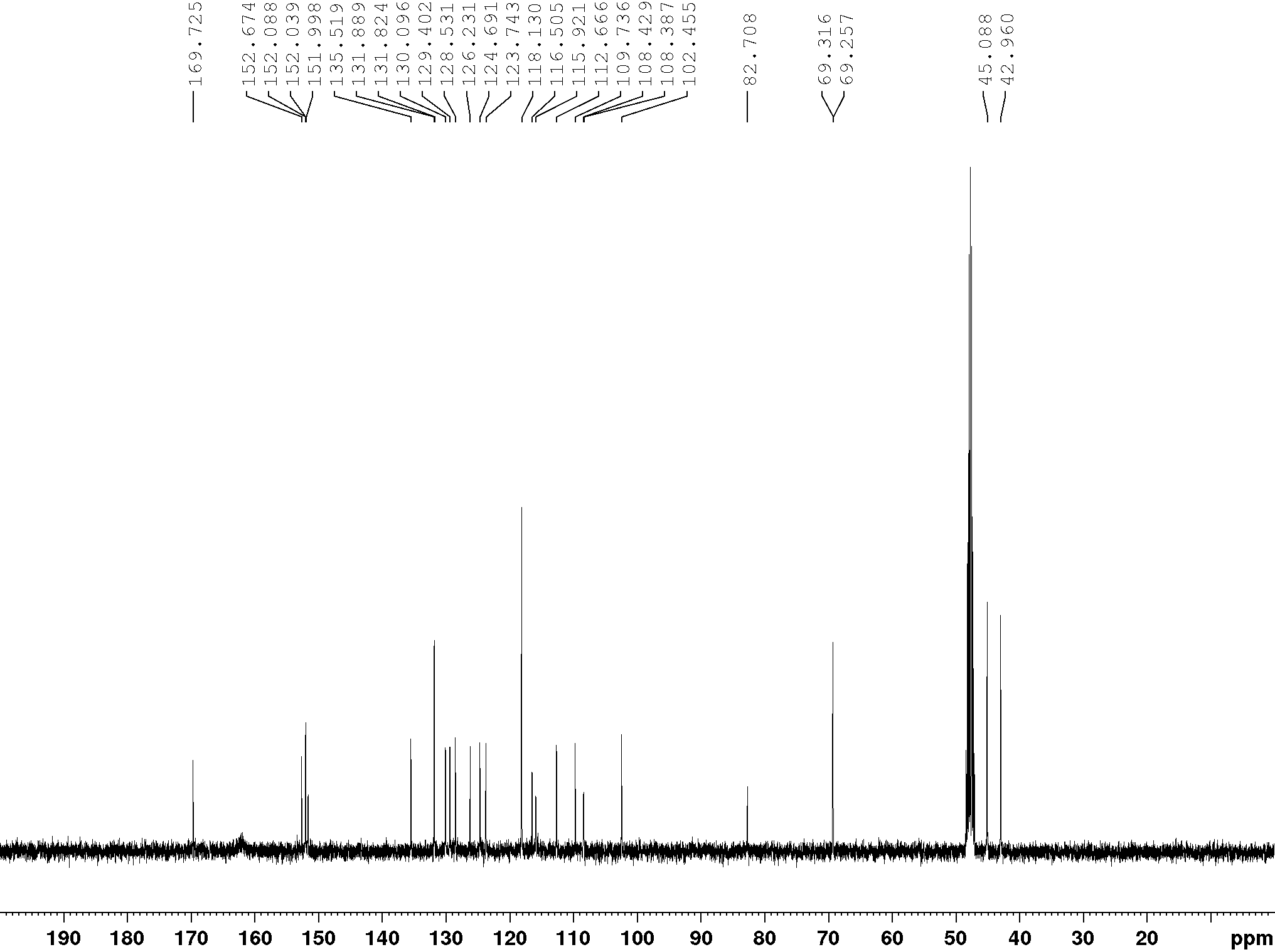


**Compound 11 (CDCl_3_, 400 MHz ^1^H NMR, 100 MHz ^13^C NMR, 162 MHz ^31^P NMR)**

**
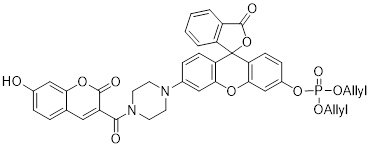
**
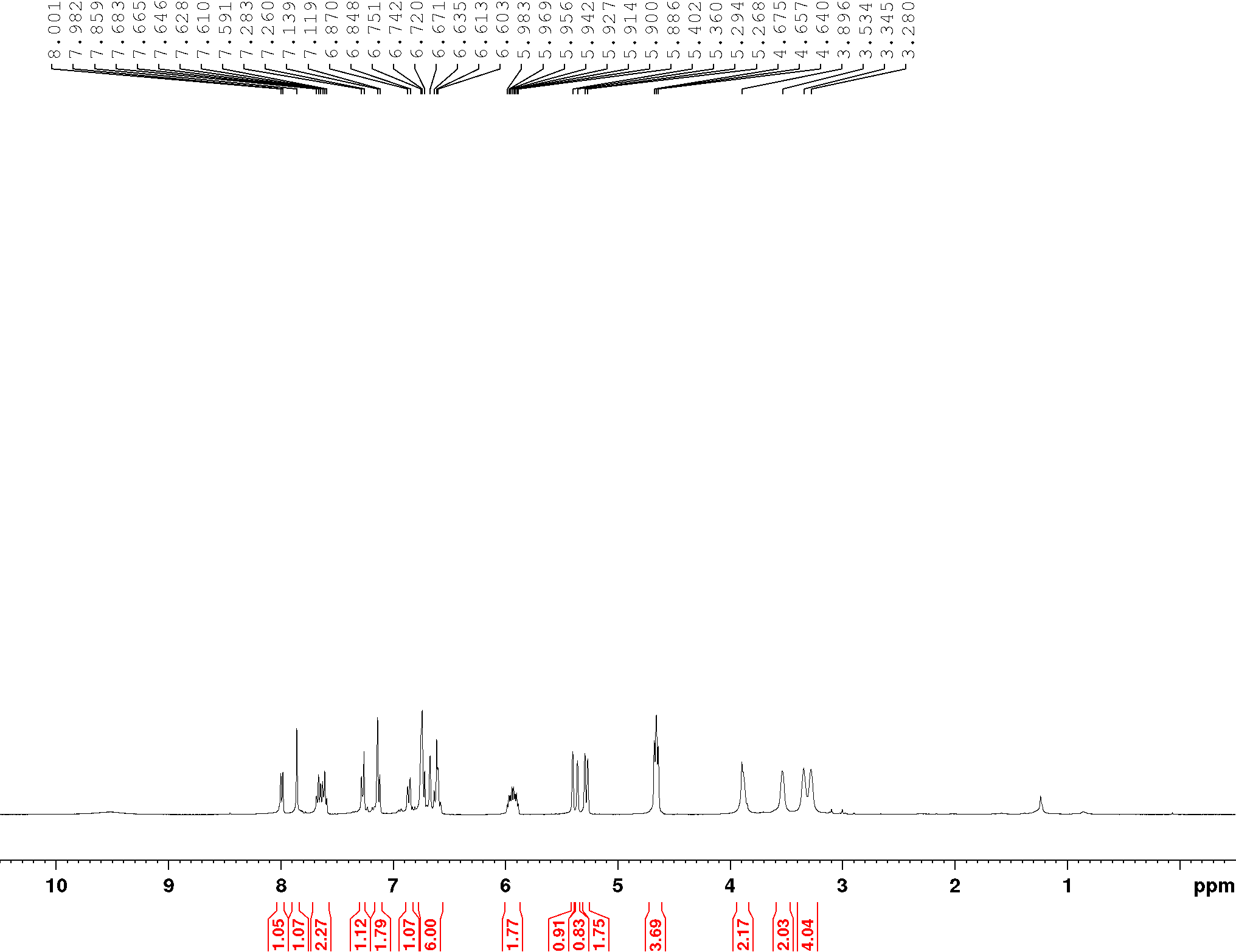


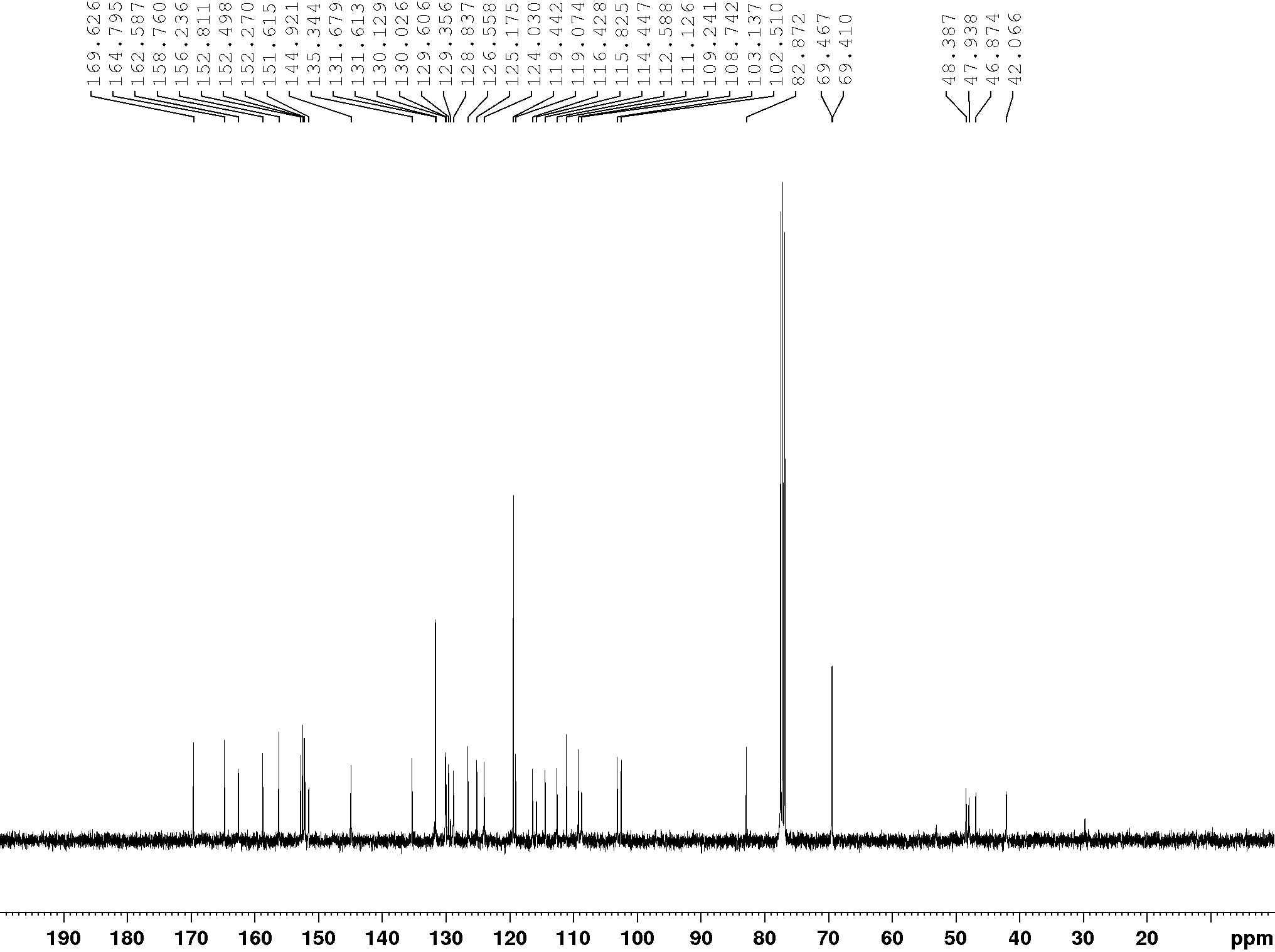


**CM-Rhod-P (DMSO-*d*_6_, 400 MHz ^1^H NMR, 100 MHz ^13^C NMR, 162 MHz ^31^P NMR)**

**
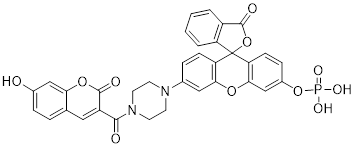
**
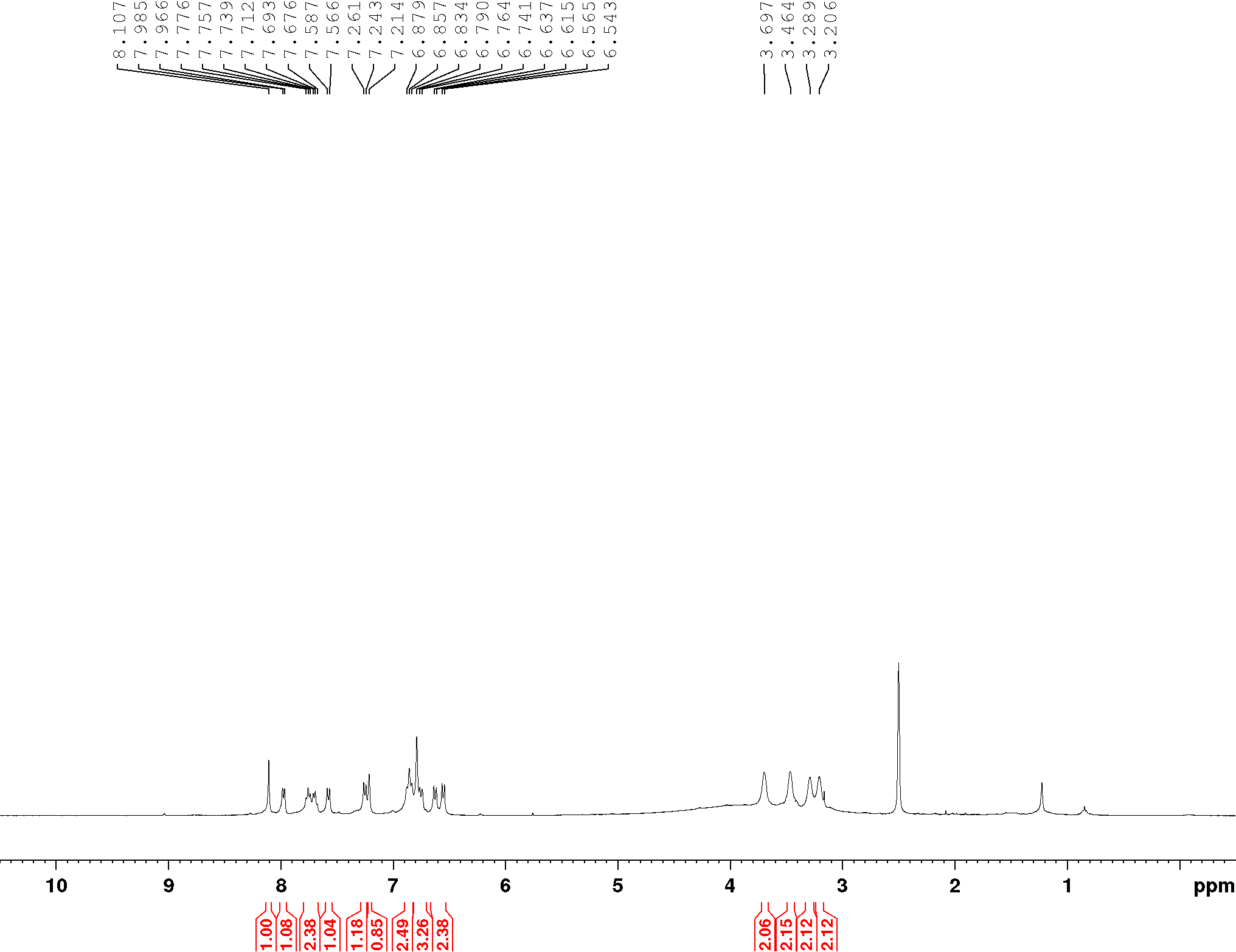


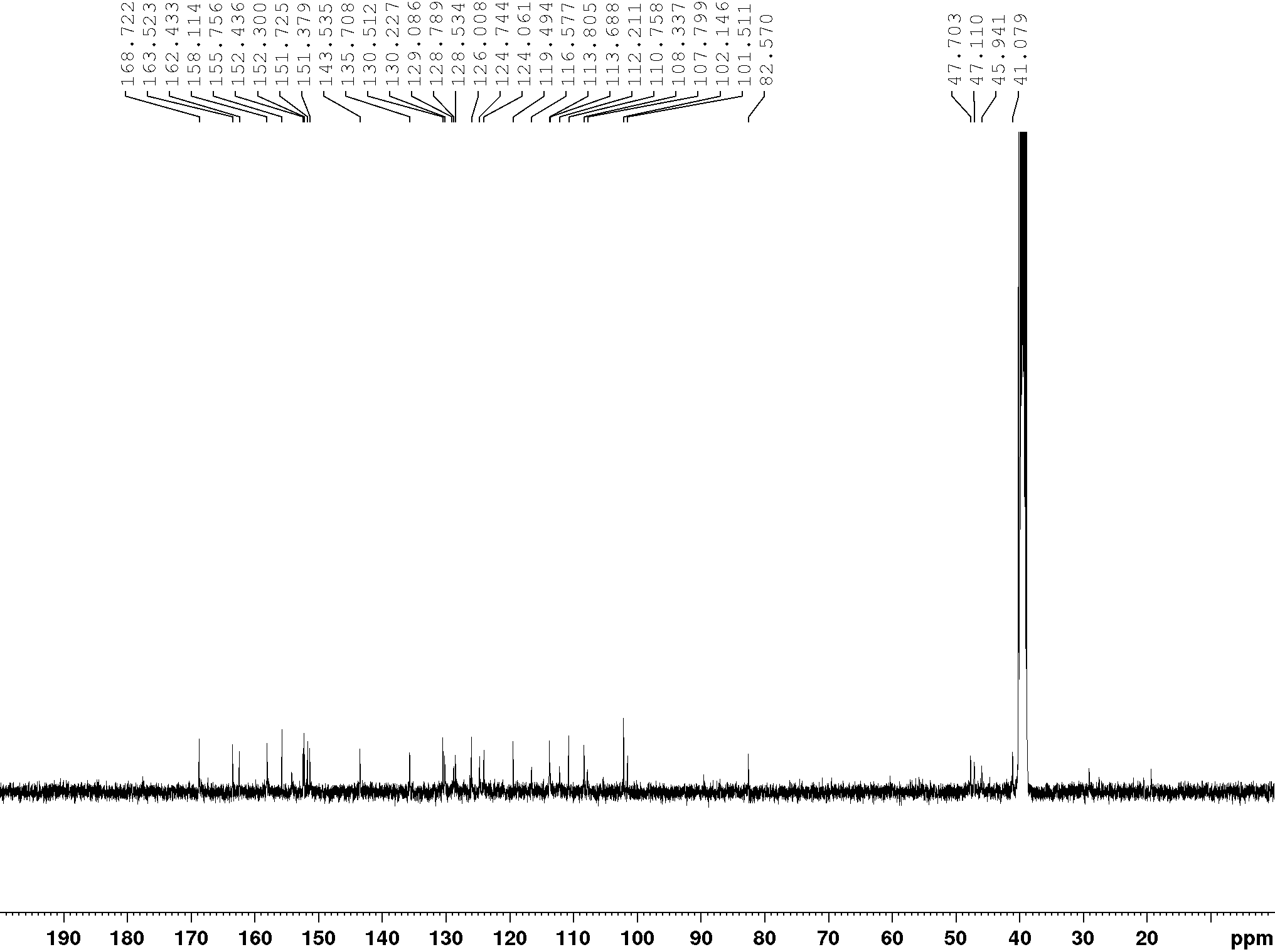


**Compound 12 (CDCl_3_, 400 MHz ^1^H NMR, 100 MHz ^13^C NMR, 162 MHz ^31^P NMR)**

**
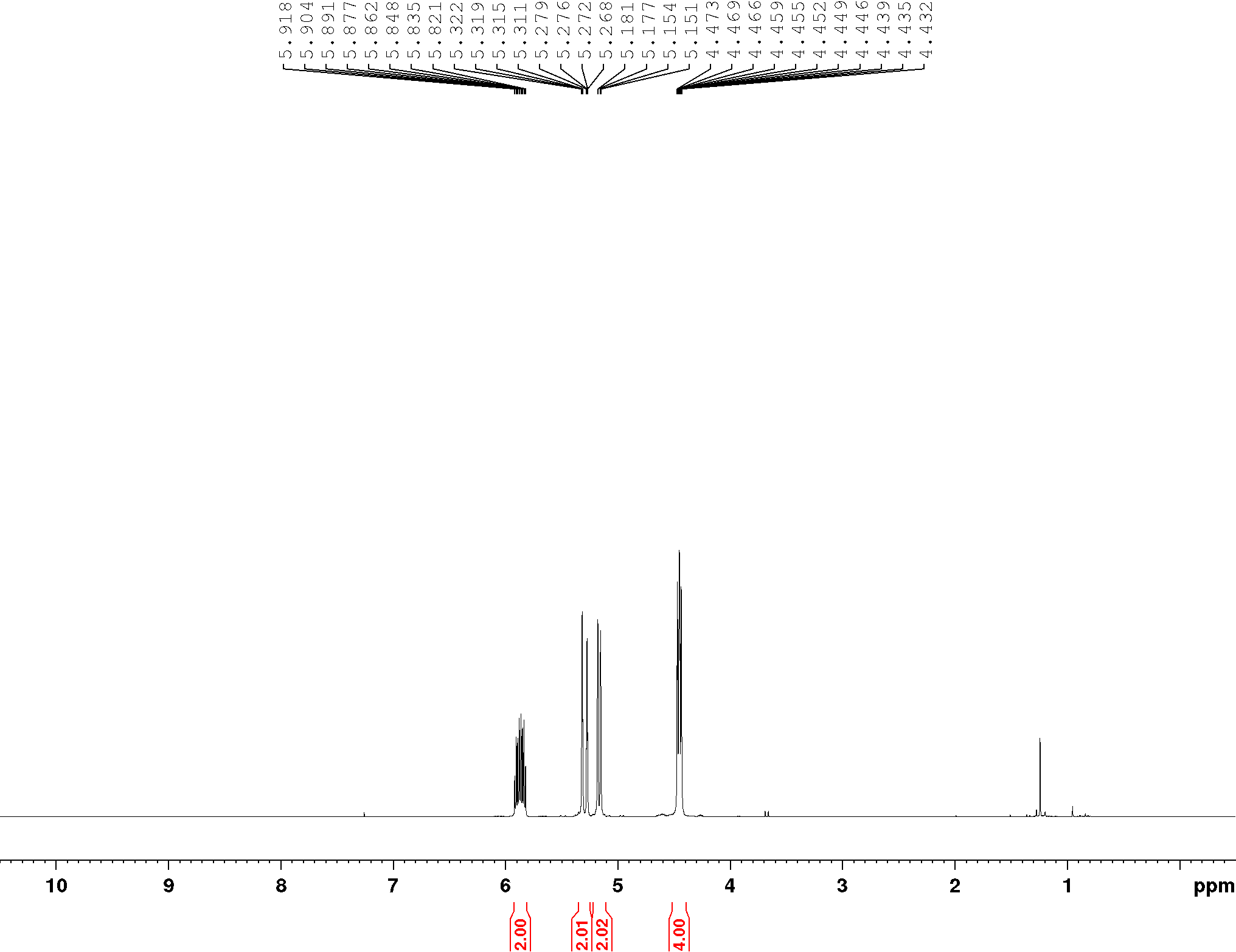
**

**
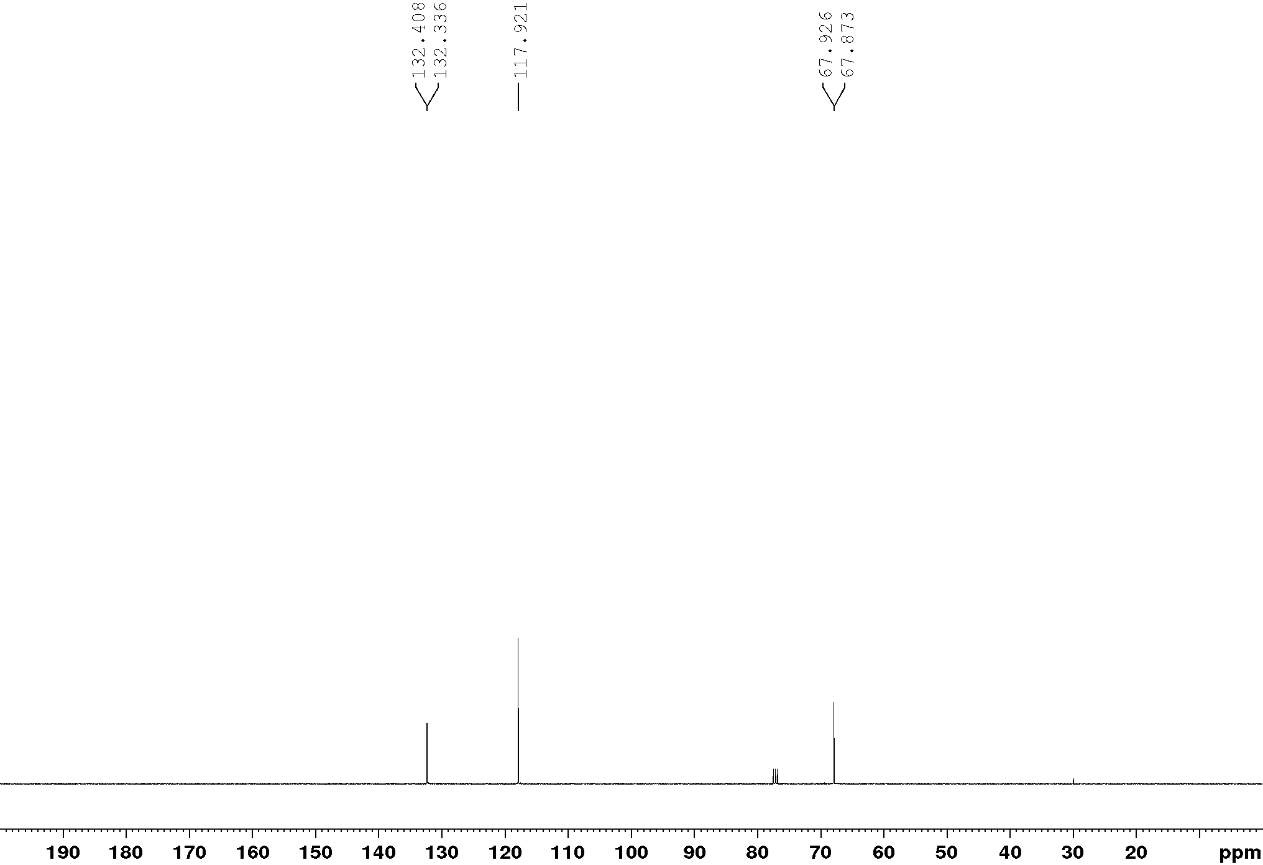
**

**
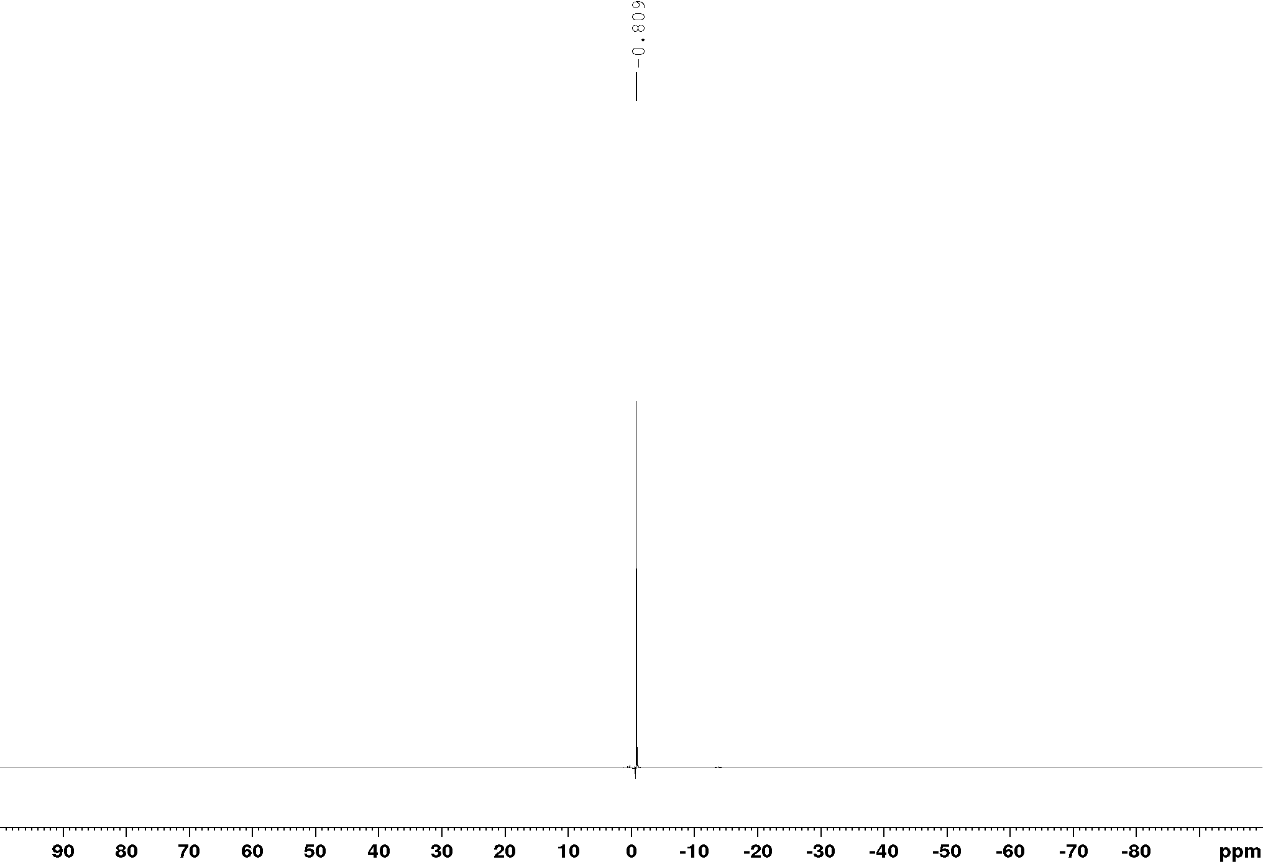
**

**Diallyl phosphoryl chloride (CDCl_3_, 400 MHz ^1^H NMR, 100 MHz ^13^C NMR, 162 MHz ^31^P NMR)**

**
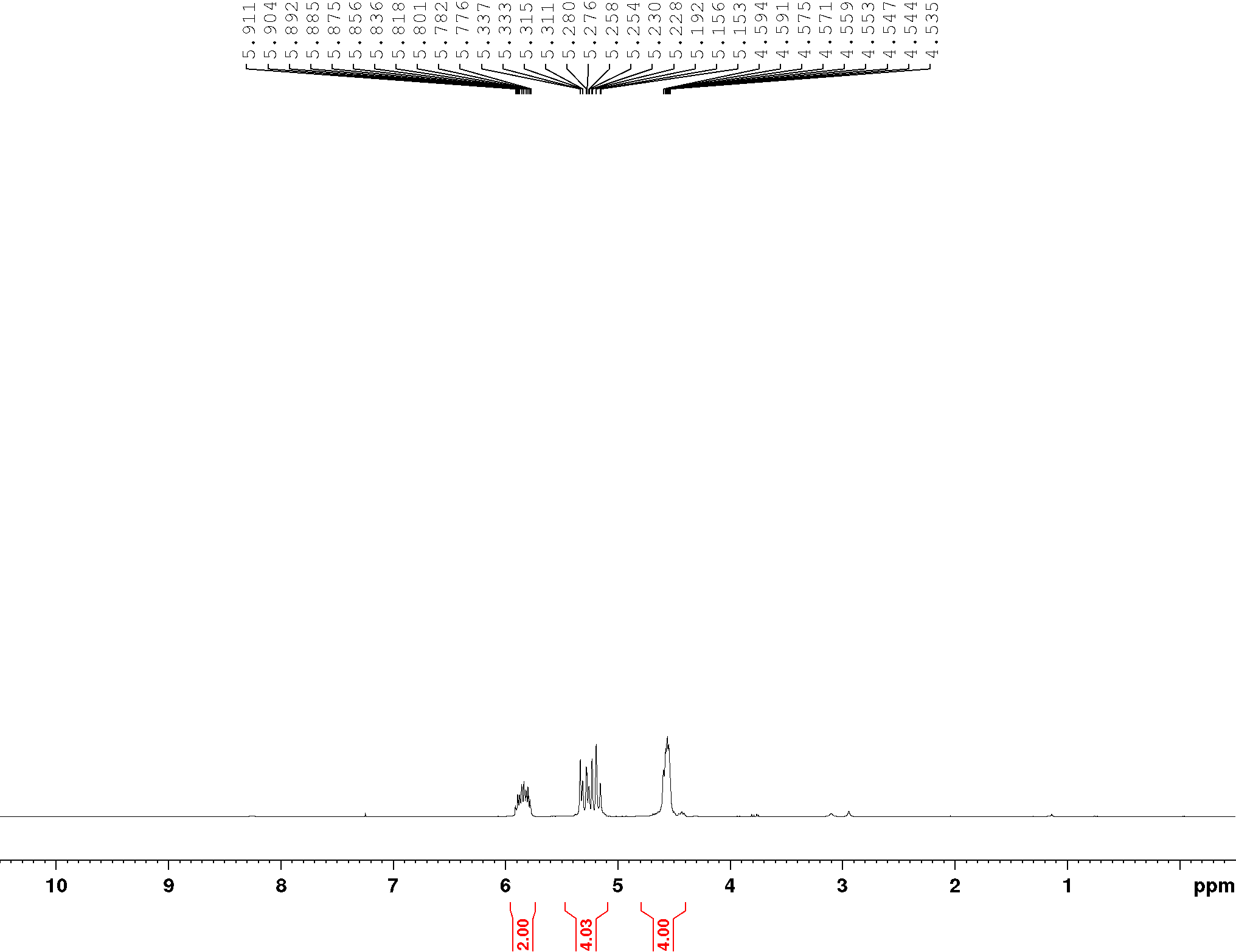
**

**
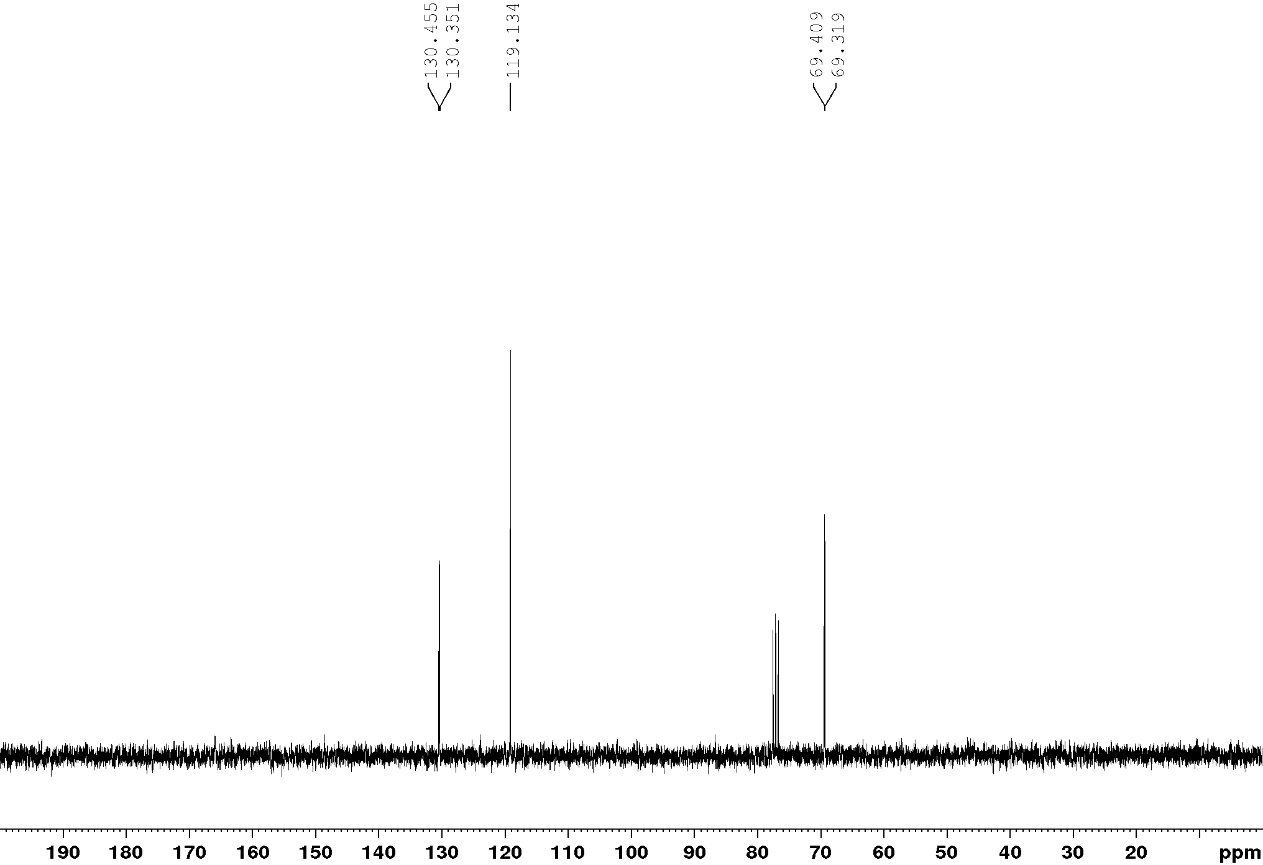
**

**
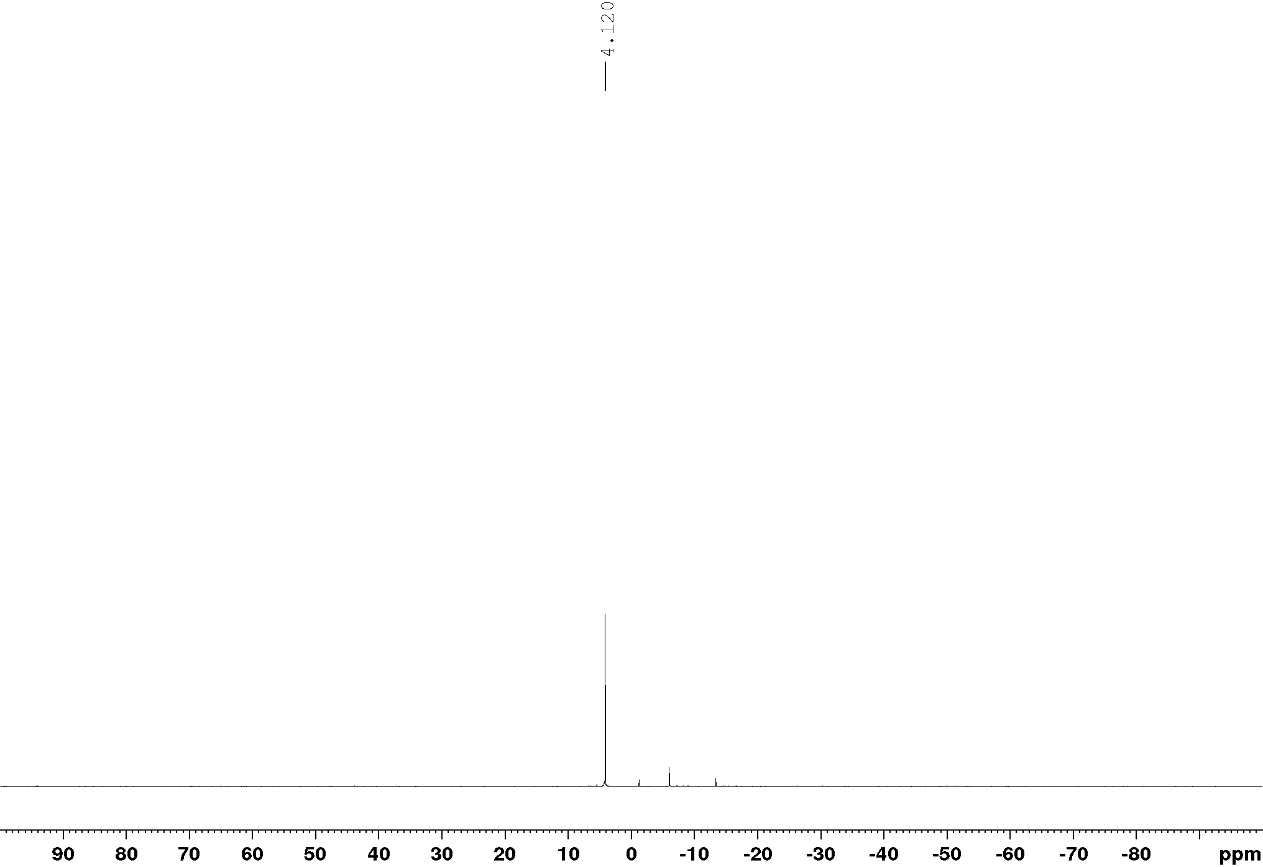
**

#

# 7. References

T. Li, A. Tikad, A. W. Pan, S. Vincent. β-Stereoselective phosphorylations applied to the synthesis of ADP- and Polyprenyl-β-Mannopyranosides, Org. Lett. 16 (2014) 5628−5631. doi:10.1021/ol5026876
